# Proteases influence colony aggregation behavior in *Vibrio cholerae*

**DOI:** 10.1016/j.jbc.2023.105386

**Published:** 2023-10-26

**Authors:** Tyler C. Detomasi, Allison E. Batka, Julie S. Valastyan, Molly A. Hydorn, Charles S. Craik, Bonnie L. Bassler, Michael A. Marletta

**Affiliations:** 1 Department of Chemistry, University of California, Berkeley, Berkeley, California, USA; 2 Department of Pharmaceutical Chemistry, University of California, San Francisco, San Francisco, California, USA; 3 Department of Molecular Biology, Princeton University, Princeton, New Jersey, USA; 4 The Howard Hughes Medical Institute, Chevy Chase, Maryland, USA; 5 Department of Microbiology and Immunology, College of Physicians and Surgeons, Columbia University, New York, New York, USA; 6 California Institute for Quantitative Biosciences, University of California, Berkeley, Berkeley, California, USA; 7 Department of Molecular and Cell Biology, University of California, Berkeley, Berkeley, California, USA

**Keywords:** proteolysis, *Vibrio cholerae*, biofilm, aggregation

## Abstract

Aggregation behavior provides bacteria protection from harsh environments and threats to survival. Two uncharacterized proteases, LapX and Lap, are important for *Vibrio cholerae* liquid-based aggregation. Here, we determined that LapX is a serine protease with a preference for cleavage after glutamate and glutamine residues in the P1 position, which processes a physiologically based peptide substrate with a catalytic efficiency of 180 ± 80 M^-1^s^-1^. The activity with a LapX substrate identified by a multiplex substrate profiling by mass spectrometry screen was 590 ± 20 M^-1^s^-1^. Lap shares high sequence identity with an aminopeptidase (termed *Vp*AP) from *Vibrio proteolyticus* and contains an inhibitory bacterial prepeptidase C-terminal domain that, when eliminated, increases catalytic efficiency on leucine *p*-nitroanilide nearly four-fold from 5.4 ± 4.1 × 10^4^ M^−1^s^−1^ to 20.3 ± 4.3 × 10^4^ M^−1^s^−1^. We demonstrate that LapX processes Lap to its mature form and thus amplifies Lap activity. The increase is approximately eighteen-fold for full-length Lap (95.7 ± 5.6 × 10^4^ M^−1^s^−1^) and six-fold for Lap lacking the prepeptidase C-terminal domain (11.3 ± 1.9 × 10^5^ M^−1^s^−1^). In addition, substrate profiling reveals preferences for these two proteases that could inform *in vivo* function. Furthermore, purified LapX and Lap restore the timing of the *V. cholerae* aggregation program to a mutant lacking the *lapX* and *lap* genes. Both proteases must be present to restore WT timing, and thus they appear to act sequentially: LapX acts on Lap, and Lap acts on the substrate involved in aggregation.

Bacteria form community aggregates that aid their survival in hostile environments (1), including those encountered during pathogenesis (2, 3). In particular, *Vibrio cholerae*, the bacterium that causes the cholera disease, exhibits two modes of community formation: surface-associated biofilms and liquid-based aggregates. Both pathways are controlled by quorum sensing, a cell-to-cell communication process (4, 5, 6). Specifically, at low cell density, *V. cholerae* forms surface biofilms, dispersing from them at high cell density. Multiple matrix components are required for surface biofilm formation. By contrast, the *V. cholerae* liquid aggregation program occurs at high cell density and does not rely on canonical biofilm matrix components. Since *V. cholerae* strains that form aggregate communities are more virulent than those that cannot, it is hypothesized that liquid aggregate formation is crucial for *V. cholerae* to successfully transit between the marine niche and the human host (7, 8, 9). Thus, understanding the molecular mechanisms underlying aggregation could enable development of new therapeutics. At present, little is known about the high cell density, liquid-based *V. cholerae* aggregation mechanism. Bacterial aggregation often involves an outer-membrane autotransporter that self-associates and can contain protease domains (10, 11, 12). For example, Hap, a nonpilus aggregation protein from *Haemophilus influenzae*, contains a protease domain at the N-terminus followed by a β-helix that is embedded in the bacterial outer membrane which oligomerizes to promote aggregation. A catalytically inactive Hap variant increases aggregation, suggesting that Hap plays a repressive role in aggregation (13). Proteases residing at the N-termini of autotransporters have also been identified in *Escherichia coli* (14). The mode of activation in *H. influenzae* is the opposite of what is observed with *V. cholerae* where proteolytic activity suppresses aggregation in the former and facilitates it in the latter. Nonetheless, the Hap example demonstrates that proteolytic activity can regulate many aspects of bacterial aggregation formation.

In *V. cholerae*, four proteases are involved in the liquid-based aggregation program: HapA, PrtV, LapX, and Lap (15). All four proteases are secreted. A mutant *V. cholerae* strain lacking all four proteases displays a greater delay in the onset of the aggregation phenotype than a mutant lacking only Lap and LapX (15) (all further mentions of Lap and LapX refer to the *V. cholerae* proteins). HapA and PrtV are known to have other roles in *Vibrio* biology, including degradation of mucin during invasion (16), proteolysis of host proteins (17), and degradation of GbpA (18), a polysaccharide monooxygenase involved in both mucin binding and chitin oxidation (19, 20). However, involvement in aggregation is the first and only identified physiological role for LapX and Lap and represents a new molecular mechanism underlying posttranslational regulation of bacterial aggregation (4, 15).

The substrate (or substrates) and the functions that LapX and Lap fulfill in the aggregation phenotype are unknown, as the insoluble nature of the aggregates has precluded substrate identification. Of the two, more can be speculated about Lap since it shares relatively high sequence identity (67%) with a protein called *Vp*AP that has previously been characterized from *Vibrio proteolyticus* (previously named *Aeromonas proteolytica*). The *Vp*AP protein, a thermostable di-zinc protease, has been studied extensively (21, 22, 23, 24, 25). LapX is a predicted to be a serine protease as it contains a conserved catalytic triad. Herein, we present a biochemical characterization of LapX and Lap. We demonstrate that LapX processes Lap to a mature form. Further, exogeneous addition of both proteins to an aggregation-defective *V. cholerae* strain that lacks the *lapX* and *lap* genes restores the WT aggregation program. Using LapX and Lap protein constructs lacking critical domains shows that both proteins are required to rescue aggregation, consistent with a protease cascade model in which LapX must process Lap in order for Lap to be functional in the aggregation process.

## Results

### Bioinformatic analysis of LapX and Lap

**Figure 1:**
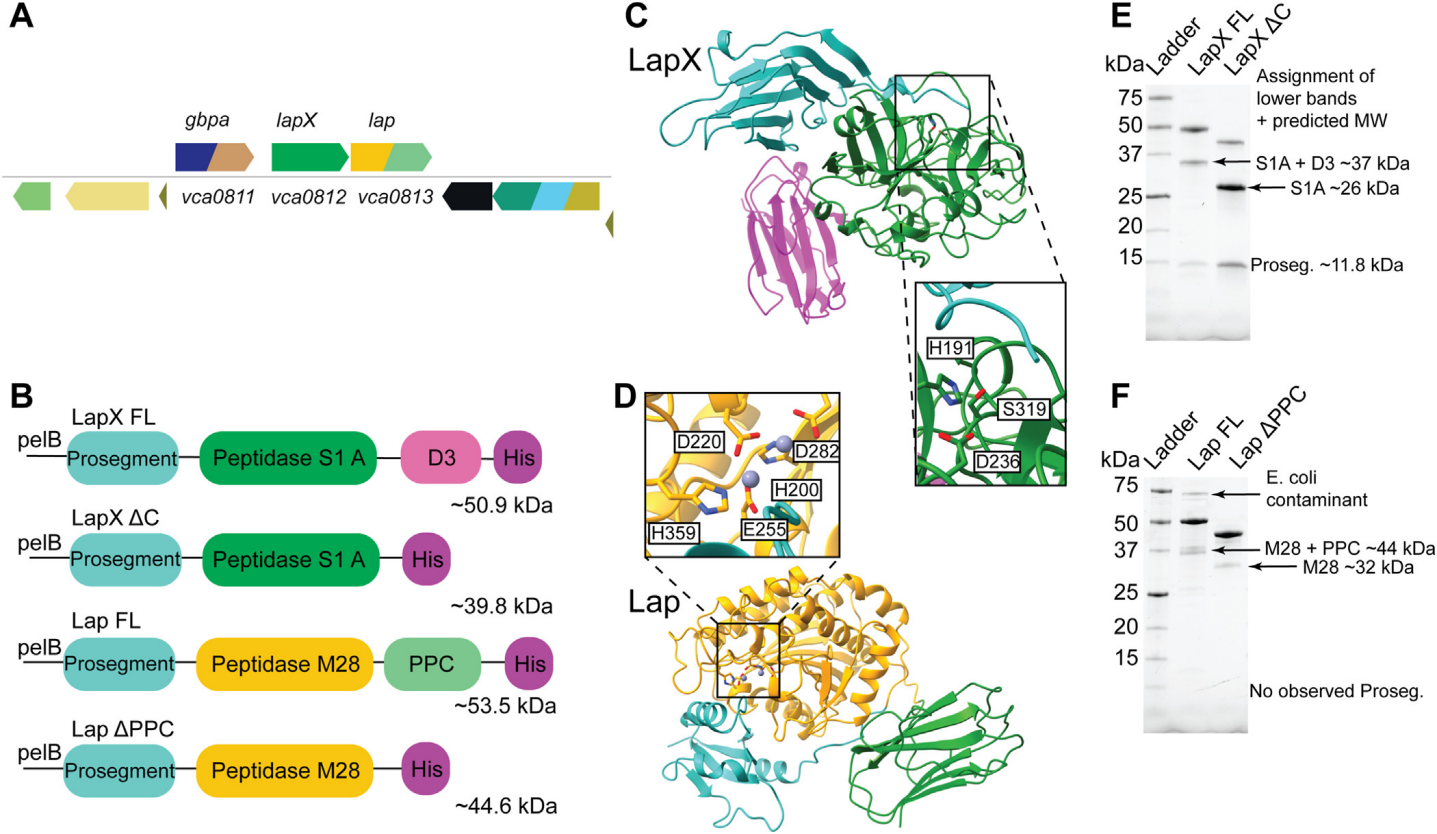
**Genetic organization, predicted structure, and purification of LapX and Lap.***A*, organization of gbpA, lapX, and lap genes. Colors represent different annotated domains. *Green*: peptidase S1 A. *Gold*: peptidase M28. *Light green*: bacterial prepeptidase C-terminal domain (PPC). *Navy*: AA10 PMO. *Tan*: chitin CBM. *B*, constructs used in this study. All contain the pelB signal peptide. *Vibrio cholerae* LapX has three domains including an N-terminal pro-segment (*cyan*), a predicted serine protease in the S1 A family (*green*), and one unannotated domain called D3 (*pink*). *V. cholerae* Lap has three domains including a prosegment (*cyan*), a metalloprotease M28 family domain (*gold*), and a prepeptidase C-terminal domain (designated PPC, *light green*). All constructs were expressed with C-terminal poly-histidine tags (designated His, *purple*). The annotated weight is the predicted protein molecular weight after signal peptide cleavage. *C*, AlphaFold2 prediction for LapX. An N-terminal prosegment (*cyan*) interacts with the surface of the protein near the active site of the protease domain (*dark green*). The protease domain is connected to the D3 domain (*pink*) through a disordered linker. *Inset*: an expanded view of the active site residues. *D*, AlphaFold2 and Alphafill prediction for Lap. The N-terminal prosegment (*cyan*) interacts with the active site surface of the protease domain (*gold*). The PPC domain (*light green*) is connected *via* a disordered linker (*gray*). *Inset*: an expanded view of the Zn-binding residues. Zn atoms are depicted as *violet spheres*. *E*, SDS-PAGE gels of the IMAC purification for the LapX constructs. The bands at ∼37 kDa and ∼26 kDa in the LapX gels correspond to the protease domains of the fully mature LapX FL and LapX ΔC proteins, respectively. The lowest bands at ∼ 15 kDa correspond to the cleaved prodomains and are the same for both proteins. The intensity of this band relative to the higher band increases throughout purification or if the protein is allowed to incubate at or above 4 °C. *F*, SDS-PAGE gels of the IMAC purification for the Lap constructs. Lower bands for each construct correspond to mature domains, ∼37 kDa and for FL and ∼30 kDa for Lap ΔC respectively, that increase in intensity if the protein is allowed to incubate at or above 4 °C. FL, full length; PPC, prepeptidase C-terminal domain.

The *vca081*2 and *vca0813* genes are located adjacent to one another in an operon on the *V. cholerae* chromosome and encode the multidomain proteins LapX (Uniprot KB Q9KLD4) and Lap (Lap, Uniprot, KB: Q9KLD3; EC 3.4.11.10), respectively (15) (Fig. 1, *A* and *B*). The genes lie downstream of *vca0811*, which encodes GbpA (Fig. 1*A*). Previous work showed that while LapX and Lap are required for *V. cholerae* aggregation, GbpA is not (15); hence we focus here on LapX and Lap. LapX is a leucine aminopeptidase-related protein, a putative serine protease that belongs to the subfamily A of the S1 family of the PA clan proteases. A search in MEROPS (MER0216699) reveals no close homologs; however, alignment of this amino acid sequence with other characterized serine proteases reveals conservation of the catalytic triad Ser, His, and Asp (Fig. S1*A*). Lap is a leucine aminopeptidase. Previously, Lap was partially purified and shown to have hydrolytic activity on a model leucyl-aminopeptidase substrate (26). A close homolog that shares 67% sequence identity, *Vp*AP, has been thoroughly characterized through biochemical and structural studies (21, 22, 23, 24, 27). *Vp*AP binds two Zn^2+^ atoms in the active site and exhibits aminopeptidase activity, including activity on leucyl substrates (28). The amino acids that bind Zn^2+^ in *Vp*AP are conserved in Lap and highlighted in Fig. S1*B*, suggesting that Lap is likely a dizinc aminopeptidase.

Both proteases were subjected to a sequence similarity network (SSN) analysis (Figs. S2*A* and S3*A*). Since other *Vibrio* species such as *Vibrio harveyi* exhibit a liquid-based aggregation behavior analogous to that in *V. cholerae* (15), the *Vibrio* genus was explored to identify homologs of LapX. Increasing the alignment score (130) generated a cluster that contained only *Vibrio* proteins. Examination of the neighboring genes in the chromosomes reveals that an operon encoding the two proteases is conserved across some species but not all (*e.g*., in the *V. proteolyticus* genome, *lapX* does not reside in close proximity to *lap*) (Fig. S2, *B* and *C*). SSN analysis of Lap reveals additional homologs and produces a larger cluster that suggests higher conservation across species (Fig. S3*A*). The genome network analysis program shows that the second largest group of domains located near *lap* genes in bacterial genomes specify another protease domain (Fig. S3*B*). It may be that many Lap homologs require processing by a partner endopeptidase for full activity (see results below) or are members of proteolytic cascades.

Lap consists of an N-terminal prosegment preceding an M28 metalloprotease domain followed by a prepeptidase C-terminal domain (PPC) (Fig. 1*B*). The PPC is presumably removed during protease maturation as reported by Honma *et al.* (26). AlphaFold2 was used to predict the structures of the two protease domains and to model the respective active sites; AlphaFill was used to model the Zn in the Lap model (29, 30). The LapX AlphaFold2 model predicts an interaction between the N-terminal prosegment and the catalytic domain. The catalytic domain exhibits a double beta barrel–like structure followed by two separate domains similar to fibronectin folds connected by an unstructured linker (Fig. 1*C*). An enlarged view of the putative LapX active site is shown highlighting the predicted catalytic triad residues, H191, D236, and S319, which are in close proximity (Fig. 1*C*). The protease domain model of Lap closely matches the X-ray structure of the *Vp*AP protein (Fig. S4). A linker connects the Lap PPC domain to the M28 domain. The enlarged view of the active site shows the positioning of the predicted Zn-binding residues, H200, D220, E255, D282, and H359 (Fig. 1*D*). Of note, the N-terminal prosegments of both proteins interact with their protease domains near the respective active sites. These regions likely stabilize protein folding during translation, similar to other proteases (31), and are removed to allow substrate access to the active sites.

### LapX and lap constructs

Both LapX and Lap contain a secretion sequence and are secreted physiologically. Various LapX and Lap constructs were generated in the pET22b plasmid where the native secretion sequence was replaced with a pelB leader sequence for transport to the periplasm. The constructs retained the same amino acids in the native zymogen and included a C-terminal polyhistidine tag for ease of purification. The full-length (FL) constructs contained all domains as shown in Figure 1*B*. To test the roles of the C-termini in protease activity, a Lap construct lacking the pre-peptidase C-terminal domain (Lap ΔPPC) was made, along with a LapX construct lacking the unannotated C-terminal domain (LapX ΔC). SDS-PAGE gels for the FL and truncated constructs for LapX and Lap are shown in Figure 1, *E*, and *F*, respectively. The smaller bands observed during purification are attributed to processing by *E. coli* proteases or, in the case of LapX, slow autocleavage leading to maturation. We have putatively assigned these bands according to approximate sizes of domains and prosegments. Catalytically inactive S319A variants of LapX and LapX ΔC were also made as controls for some assays.

### Characterization of Lap

**Figure 2:**
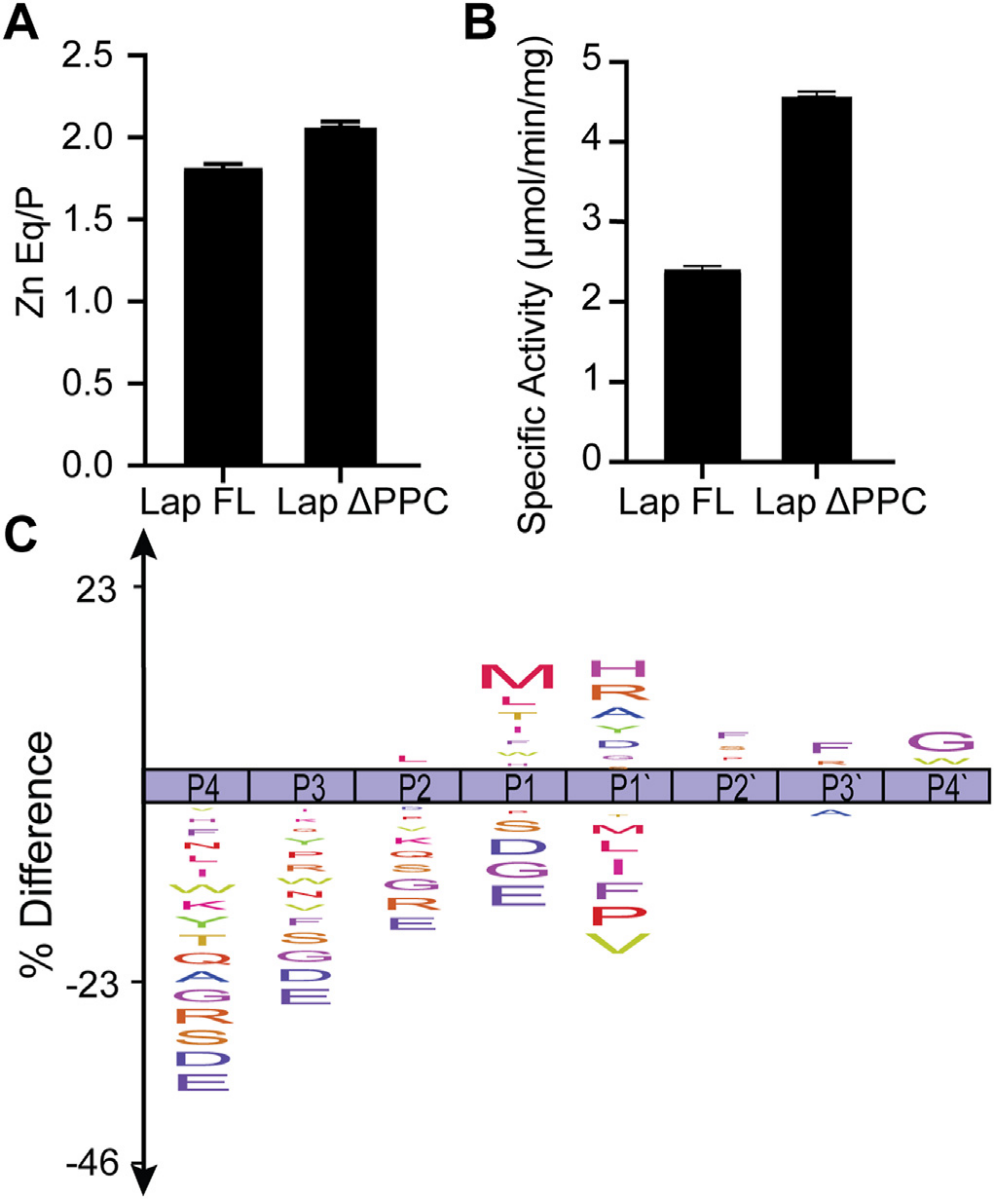
**Characterization of Lap.***A*, zinc equivalents for Lap protein constructs determined by ICP. Zinc content is ∼1.8 and ∼2.1 Zn^2+^ equivalents/protein (Eq/P) for Lap FL and Lap ΔPPC, respectively. Zinc measurements were performed in technical triplicate, and error bars represent SEM. *B*, activity of Lap FL and Lap ΔPPC on the aminopeptidase model substrate, leucyl-*p*-nitroanilide. Assays were performed in biological triplicate, and error bars represent SEM. *C*, Lap FL ICE logo representing consolidated MSP-MS data. These data show a pattern consistent with amino protease activity that cleaves preferentially at Met, Leu, Thr, Ile in the P1 position, with a secondary preference for His, Arg, Ala, Tyr in the P1′ position. FL, full length; MSP-MS, multiplex substrate profiling-mass spectrometry.

Purified Lap FL and Lap ΔPPC contain ∼1.8 and ∼2.1 equivalents of Zn^2+^, respectively (Fig. 2*A*), mirroring the two equivalents observed for its homolog *Vp*AP. Both constructs were active on the model substrate for leucyl aminopeptidases, leucyl *p*-nitroanilide. The Lap ΔPPC truncation exhibited 2-fold higher specific activity than Lap FL (Fig. 2*B*). This finding is consistent with previous reports of Lap (26) and its homolog *Vp*AP (27), which exhibit higher protease activity when processed.

To define Lap substrate preferences, multiplex substrate profiling-mass spectrometry (MSP-MS) was used to compare Lap FL and Lap ΔPPC (Figs. 2*C*, and S5*A*). MSP-MS exploits a chemically diverse peptide library to determine protease substrate preference (32). MSP-MS is a useful technique to provide information on the class of peptidase (*i.e.*, endo-, amino-, or carboxy-peptidase) and general specificity for tested proteases. The MSP-MS peptide library excludes cysteine from any position in the library and only considers the amino acids of all neighbor and near neighbor amino acid pairs. Additionally, for exopeptidases, there are only 228 aminopeptidase substrates and 228 carboxypeptidase substrates. Nonetheless, results from this data set allow for identification of a model substrate for comparison of protease behavior and could be potentially leveraged to identify native substrates. However, since it is an artificial library and not all of peptide space, all potential cleavages from the protease itself are not observed, and therefore results may not aid in the identification of physiological substrates. This method is also semiquantitative as each individual peptide and subsequent cleavage products have their own ionization properties. Thus, MSP-MS was used to probe the peptide substrate specificity of both the two Lap constructs and two LapX constructs and to investigate the general peptidase class *e.g.* endo-, amino-, or carboxy- etc. A table with the top observed cleavages for each replicate is presented (File S1). Both Lap protein constructs exhibited aminopeptidase activity with a cleavage preference for N-termini in the peptidase library. The Lap constructs exhibit specificity for hydrophobic residues or Thr at the substrate P1 position. This cleavage pattern indicates aminopeptidase activity as the P1 position is the N-terminal residue. We observed both mono- and diaminopeptidase cleavage where the mono or di refers to the number of residues on the N-terminal side of cleavage. Lap FL and Lap ΔPPC showed the highest selectivity for substrates with Met (modeled by norleucine in the MSP-MS library) at the P1 position. Leu was the second most preferred residue at the P1 site for Lap FL and the third highest for the Lap ΔPPC construct. There are very minor differences in substrate preferences between the two constructs.

### Initial characterization of LapX

**Figure 3:**
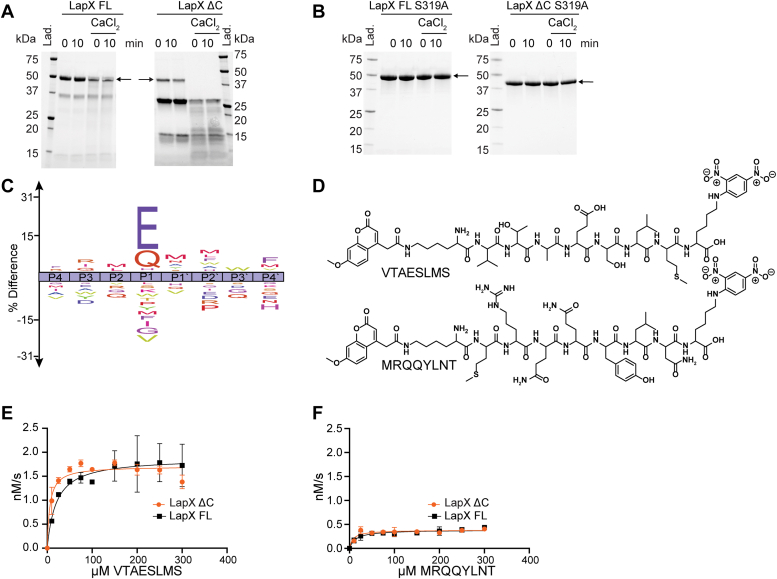
**Characterization of LapX.***A*, the effect of Ca^2+^ on LapX and LapX ΔC autoprocessing with instant quenching (0 min) and after 10 min at RT (10 min). *B*, LapX FL S319A and LapX ΔC S319A do not autoprocess with instant quenching (0 min) and after 10 min at RT (10 min) even in the presence of Ca^2+^. *C*, LapX FL ICE logo representing MSP-MS results. The most highly cleaved peptides, as measured by MSP-MS for LapX FL, were chosen for Glu at the P1 position (*panel E*) and Gln at the P1 position (*panel F*). *D*, structures of the internally quenched substrates used to measure activity in panels E and F with K(MCA) at the N terminus and spectrally matched K(DNP) at the C terminus of octapeptides adapted from top MSP-MS peptide results. *E* and *F*, activity of LapX FL and LapX ΔC on internally quenched peptides VTAESLMS (*E*) and MRQQYLNT (*F*). Each point was performed in biological triplicate, and error bars depict SEM. FL, full length; MSP-MS, multiplex substrate profiling-mass spectrometry.

Over the course of purification, both LapX and LapX ΔC were processed into smaller proteins with proteolytic cleavage and loss of the prosegment (∼11 kDa). While there appears to be a slow component of maturation during purification, we found LapX ΔC could rapidly autoprocess into a ∼26 kDa fragment in the presence of Ca^2+^ (Fig. 3*A*), which corresponds to the predicted S1A domain. There also appears further processing of the protein with the activation of Ca^2+^ beyond the 26 kDa fragment. Autoprocessing was not blocked by the general serine protease inhibitor, AEBSF, or other commercially available serine protease inhibitors (Fig. S6). Calcium dependence is consistent with the finding that aggregation in *V. cholerae* occurs when elevated Ca^2+^ is present (4). LapX autoprocessing was not enhanced by either zinc or magnesium (Fig. S5). The putative active site serine was changed to alanine (S319A) in LapX FL and LapX ΔC. We call these constructs LapX FL S319A and LapX ΔC S319A, respectively. These variants were purified and tested for autoprocessing activity. Neither catalytically inactive S319A variant underwent processing in the presence or absence of calcium, results that are consistent with autoprocessing events for the constructs containing the S319 residue (Fig. 3*B*).

To determine LapX substrate specificity, LapX FL and LapX ΔC were subjected to MSP-MS (32, 33). We reasoned that analysis of LapX ΔC could provide information concerning a role for the C-terminal domain in substrate preference, as this domain is unannotated and its function, if any, is unknown. However, results with the two proteins were quite similar as both exhibited endopeptidase activity and very small differences in specificity at any given position, with strong preferences for Glu and reduced preferences for Gln at the P1 position (Figs. 3*C* and S5*B*). To validate the observed specificity, an internally quenched peptide was synthesized as previously described (30), corresponding to the most highly cleaved peptide across all MSP-MS runs (VTAESLMS) (Fig. 3*D* top). Similarly, we also synthesized an internally quenched peptide substrate corresponding to the highest observed cleavage of a substrate containing a Gln at the P1 position (MRQQYLNT) (Fig. 3*D* bottom). The Ice logo represents the composite specificity of all the observed cleavages; however, this composite data does not take into account the potential for amino acid cooperativity in any particular substrate. To avoid that potential issue, these two sequences were selected; since they were already demonstrated to be competent substrates; the top 30 observed cleavages are presented in File S1. This is similar to what has been done previously with the MSP-MS platform where the top cleaved peptides were used to generate model substrates; this strategy has been shown to provide optimal substrates for other proteases (32, 33). Compared to one replicate of LapX ΔC S319A as a negative control, neither top hit was observed in the catalytically inactive serine to alanine variant (File S1). The Glu-containing peptide was hydrolyzed with *k*_cat_ values of 3.76 ± 0.39 × 10^−3^ s^−1^ for LapX FL and 3.42 ± 0.3 × 10^−3^ s^−^^1^ for LapX ΔC (Fig. 3*E*). These turnover numbers are roughly 5-fold higher than that for the peptide with Gln at the P1 position (Fig. 3*F*), with *k*_cat_ values of 7.7 ± 0.7 × 10^−4^ s^−1^ and 7.7 ± 0.6 × 10^−4^ s^−1^ for LapX FL and LapX ΔC, respectively. Additionally, there appears to be only a slight or negligible effect of Ca^2+^ on the rates of LapX FL and LapX ΔC-mediated peptide hydrolysis (Table 1).

**Table 1:** Kinetic parameters for LapX internally-quenched substrates

Substrate	Construct	*k*_*cat*_ (S^−1^)	±	*K*_*M*_ (μM)	±	*k_cat_*/*K*_*M*_ (M^−1^S^−1^)	±
MMPESIAN (Lap)	LapX FL	0.0025	0.0002	34.6	12.2	73	26
	LapX FL + Ca	0.0024	0.0004	61.1	27.3	40	19
	LapX ΔC	0.0031	0.0003	17.2	7.5	180	80
	LapX ΔC + Ca	0.0026	0.0002	15.1	6.4	171	73
VTAESLMS (MSP-MS)	LapX FL + Ca	0.00376	0.00039	20.1	9.0	187	86
	LapX ΔC + Ca	0.00342	0.00019	5.8	2.4	590	20
MRQQLYNT (MSP-MS)	LapX FL + Ca	0.00077	0.00007	14.0	6.4	55	26
	LapX ΔC + Ca	0.00077	0.00006	8.1	4.0	94	47

### LapX processing of Lap

**Figure 4:**
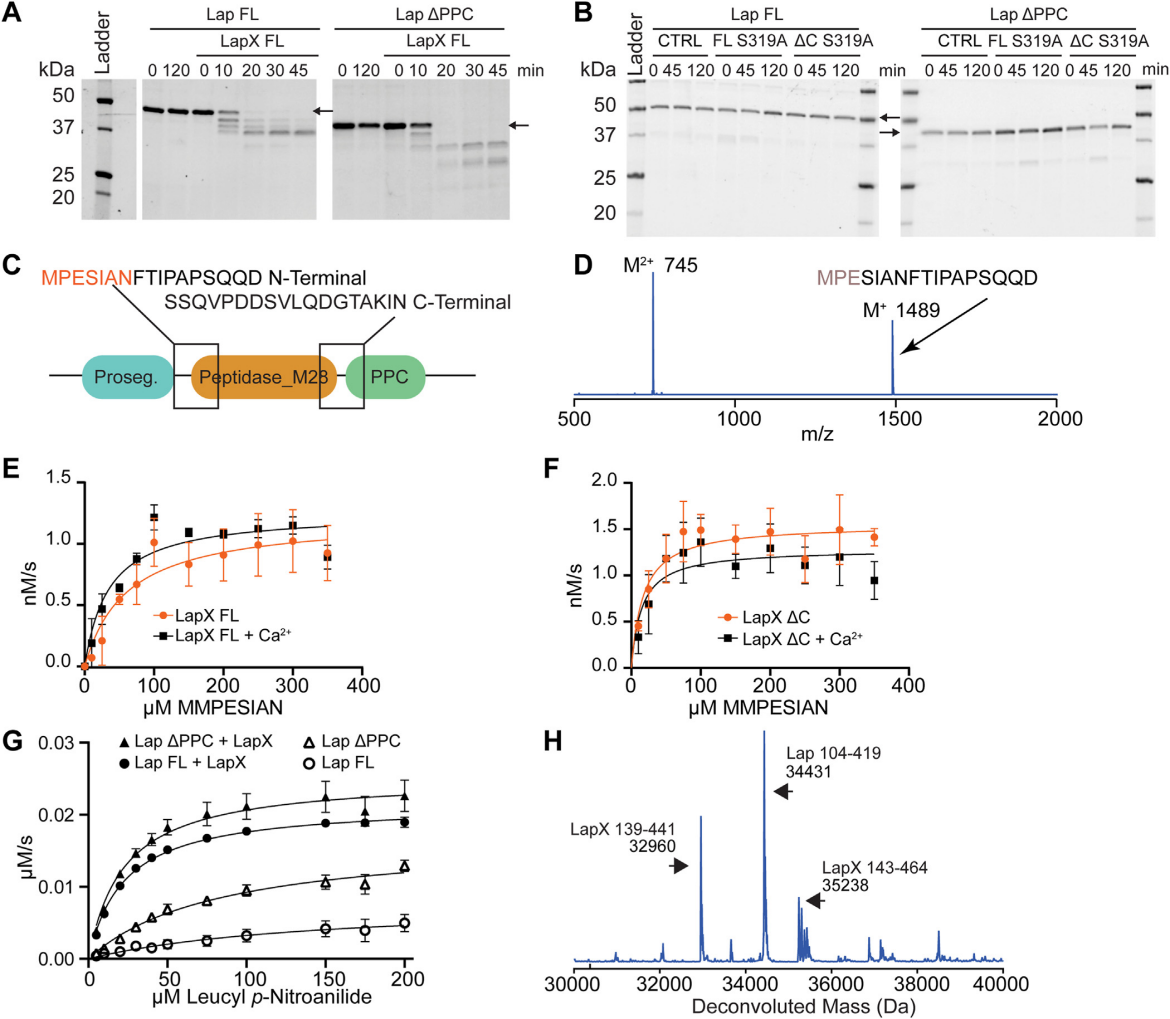
**Characterization of LapX processing of Lap.***A*, SDS-PAGE gel showing that LapX processes both Lap FL and Lap ΔPPC, and neither Lap construct autoprocesses for up to 120 min. The main ∼50 kDa Lap FL band is processed to a ∼37 kDa band. Lap ΔPPC is processed from ∼37 kDa to two smaller bands of ∼30 and ∼27 kDa. Gel has been cropped for clarity. Arrow denotes initial uncleaved Lap protein band. LapX constructs are not visible on the gel due to the low catalytic amount added. *B*, alteration of Ser 319 to alanine eliminates LapX processing of Lap. CTRL represents no LapX construct added. Arrow denotes initial uncleaved Lap protein band. LapX constructs are not visible on the gel due to the low catalytic amount added. *C*, schematic of Lap peptide substrates chosen to test LapX activity. These peptides flanked the N and C termini of the protease domain corresponding to MPESIANFTIPASQQD and SSQVPDDSVLQDGTAKIN, respectively. *D*, mass spectrum of the N-terminal Lap peptide revealing a fragment corresponding to cleavage after Glu97. *E* and *F*, activity of LapX FL and LapX ΔC, respectively, on the internally quenched FRET-based peptide substrate K(MCA)MMPESIAN K(DNP) with and without Ca^2+^. Each point was performed in biological triplicate and error bars depict SEM. *G*, kinetics of LapX activation of Lap for hydrolysis of leucyl *p*-nitroanilide. Each point was performed in biological triplicate and error bars depict SEM. *H*, whole protein mass spectrum of Lap ΔPPC treated with LapX FL. The peaks correspond to two LapX species, the peak at 32690 Da matches the Edman sequencing data that corresponds to the tetrapeptide GINE. The Lap species beginning at Ile104 matches Edman sequencing data for the pentapeptide IPAPS. FL, full length.

Previously, it was shown that proteinase K treatment of the Lap homolog *Vp*AP was sufficient to convert the aminopeptidase into its mature form (27). Since *lapX* is presumably expressed together with *lap*, it was reasonable to test if LapX acted on Lap. Purified Lap was incubated with catalytic amounts of LapX FL, and the reaction was monitored by SDS-PAGE (Fig. 4*A*). LapX FL does indeed convert Lap to a mature form. To test that this process is mediated by LapX and not by Lap itself, we tested this activity with a series of experiments that contained protease inhibitors and Ca^2+^. LapX FL processing of Lap was not inhibited by an EDTA-free protease cocktail or by EDTA at a concentration lower than that of Ca^2+^ (Fig. S7). High levels of Ca^2+^ activate LapX FL so strongly that Lap is fully degraded in these conditions. Only boiled LapX FL and EDTA at a concentration higher than that of Ca^2+^ inhibited the process (Fig. S7). EDTA binds Zn^2+^ five orders of magnitude more strongly (34) than it binds Ca^2+^. Therefore, even with Ca^2+^ present at three orders of magnitude higher than Zn^2+^ (10 mM vs. 10 μM), chelation favors Zn^2+^ removal from Lap, which would result in a loss of Lap catalytic activity. However, no loss of Lap processing occurred during co-incubation with LapX and EDTA with excess calcium. This result suggests that Lap does not autoprocess but rather LapX processes Lap. The LapX FL S319A and LapX ΔC S319A variants did not process Lap, demonstrating that Lap processing requires catalytically active LapX (Fig. 4*B*).

To obtain a physiological-like peptide substrate for kinetic studies, two peptides of around 20 amino acids each that corresponded to the N- and C-terminal regions of the Lap M28 protease domain were obtained (Fig. 4*C*) and tested individually for cleavage by LapX FL and LapX ΔC. Cleavage was only observed for the N-terminal peptide derived from the Lap sequence (Fig. 4*D*) at Glu 97. The C-terminal cleavage sequence (Fig. 4*C*) was predicted to be cleaved after Q in the sequence SVLQ/DGTA, as Q is one of the two primary amino acids accepted in the P1 position. The lack of cleavage at the C-terminus is likely due to the low efficiency of LapX on peptide substrates. Moreover, cleavage only occurred between residues that would correspond to the P1 and P1′ position of the substrates which matches the P1 and P1**′** position from the top hits in the MSP-MS experiment and the substrates that were generated for LapX. An internally quenched FRET-based peptide substrate was designed and synthesized with the addition of a residue at the N terminus and a truncation to the P4′ residue at the C-terminus. Thus, this peptide substrate contained P1′-P4′ residues, as most proteases exhibit substrate preference at these positions (35). Activity on this physiological-like peptide substrate was assessed for LapX FL and LapX ΔC. The *k*_cat_ for LapX FL was 2.5 ± 0.2 × 10^−3^ s^−1^ and 2.4 ± 0.4 × 10^−3^ s^−1^ with and without Ca^2+^, respectively (Fig. 4*E* and Table 1). The K_m_ did not appear to be Ca^2+^-dependent. The same trend was observed with LapX ΔC with both the *k*_cat_ and *K*_*M*_ values being slightly improved for LapX ΔC compared to LapX FL, mirroring the results from SDS-PAGE (Fig. 4*F* and see Table 1). Pretreatment with AEBSF inhibited the reactions (Fig. S8*A*) but only at a much higher IC_50_ (∼5 mM) than most other previously characterized proteases that are typically inhibited at ∼10 μM (36, 37). Similarly, rat anionic trypsin is not fully inhibited by the related compound, PMSF, even at 30 mM (38). The LapX FL S319A and LapX ΔC S319A protein harboring the altered active site residue exhibited no activity against both of the designed internally quenched substrates that contain an E at the P1 position (Fig. S8, *B* and *C*). This is consistent with the control MSP-MS experiment with LapX ΔC S319A where no cleavage of the peptide containing MMPRESIAN was observed. Importantly, consistent with the SDS-PAGE analysis and as mentioned above, the LapX FL S319A and LapX ΔC S319A variants are inactive in autoprocessing and Lap-processing experiments (Fig. 4*B*).

When converted to its fully mature form by LapX FL, the Lap *k*_cat_ and *K*_*M*_ increased ∼4-fold and decreased ∼4-fold, respectively (Fig. 4*G* and Table 2). The size of the processed Lap protein was determined by mass spectrometry (Fig. 4, *C* and *D*) and the N-terminal sequence by Edman sequencing. The results are consistent with previously published data for *V. cholerae* Lap and *Vp*AP (25, 26). A truncated Lap construct containing residues I104 to Q396 was designed (Fig. S9*A*) that corresponded to the cleavage data and domain boundary according to the AlphaFold predicted structure, that is, starting at the I104 residue determined by Edman sequencing and spanning to residue Q396. We observed a prominent peak in the deconvoluted whole protein mass spectrum that corresponds to I104-Q419 that includes the linker region after the catalytic domain (Fig. 4*H*). This Lap construct was expressed using the pelB system and purified using Zn-IMAC similarly to how FL and Lap ΔPPC were purified (Fig. S9*A*). This Lap construct has low Zn^2+^ content following purification and thus exhibits low catalytic activity (Fig. S9, *B* and *C*). This result demonstrates that the N-terminal prosegment is important for folding and properly loading the metallocofactors.

**Table 2:** Kinetic parameters for Lap on leucyl-*p*-nitroanilide

Substrate	Construct	*k*_*cat*_ (s^-1^)	±	*K_M_* (μM)	±	*k_cat_/K_M_*(M^-1^s^-1^)	±
L-PNA	Lap FL	8.0	2.9	148	99	5.4 × 10^4^	4.1 × 10^4^
L-PNA	Lap FL + LapX	21.6	0.3	22.5	1.3	95.7 × 10^4^	5.6 × 10^4^
L-PNA	Lap ΔPPC	17.0	1.5	83	16	20.3 × 10^4^	4.3 × 10^4^
L-PNA	Lap ΔPPC + LapX	25.3	1.1	22.3	3.6	113 × 10^4^	19.0 × 10^4^

### Exogenous addition of LapX and Lap to *V. cholerae* mutants restores aggregation

**Figure 5:**
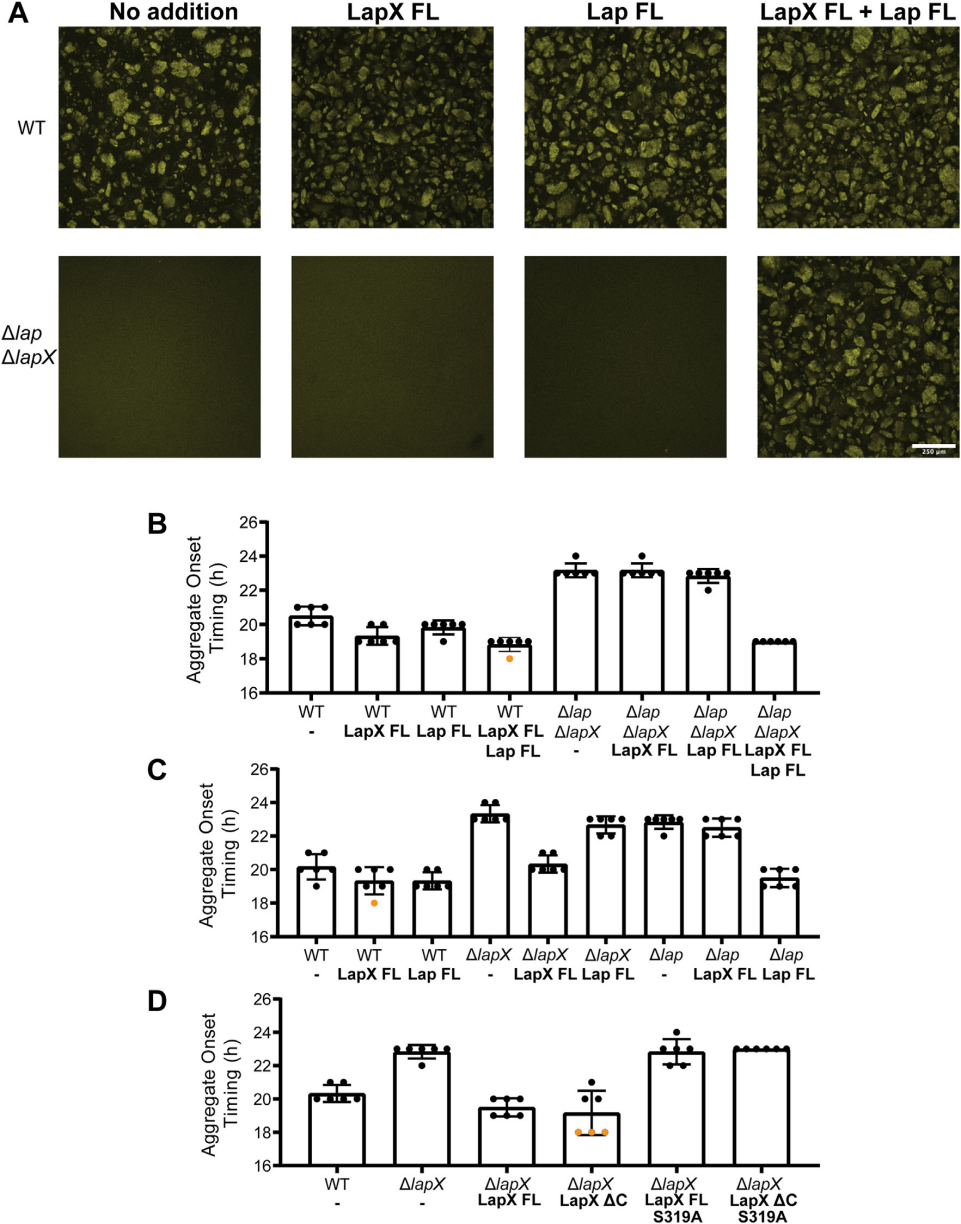
**Addition of purified LapX and Lap restore *Vibrio cholerae* aggregation in mutants lacking the proteins.***A*, fluorescent microphotographs of *V. cholerae* strains showing that deletion of both *lapX* and *lap* delays aggregation. When both LapX FL and Lap FL are added back (*bottom right*), aggregation is restored. *B*, quantitation of the data in (*A*). *C* and *D*, quantitation of aggregate formation timing in the designated strains following the designated protein additions. In (*B*–*D*), n = 6 biological replicates, error bars represent SD. Added proteins shown in *bold* print. Any sample that showed aggregation prior to 18 h is denoted as an *orange* dot, as these samples have undergone aggregation prior to the first image taken at 18 h. FL, full-length.

LapX and Lap influence the timing of *V. cholerae* aggregation (15). Here, the ability of exogenously administered LapX and/or Lap protein to restore proper timing to aggregate formation was tested using *V. cholerae* mutants. Deletion of *lapX* and/or *lap* causes a delay in aggregation (15) (Fig. 5, *A* and *B*). Addition of LapX FL to a Δ*lapX* mutant and Lap to a Δ*lap* mutant complemented the aggregate formation timing defects (Fig. 5*C*). The proteases cannot substitute for one another, as shown by the addition of Lap to the Δ*lapX* mutant and addition of LapX FL to the Δ*lap* mutant (Fig. 5*C*). Moreover, supplementation with both LapX FL and Lap was required to restore WT aggregation timing to the Δ*lapX* Δ*lap* double mutant (Fig. 5, *A* and *B*). Thus, both proteases are required for proper timing of aggregate formation. As previously reported (15), deletion of *gbpA*, the gene immediately upstream of *lapX* and *lap* on the *V. cholerae* chromosome, does not affect aggregate formation timing (Fig. S10). Likewise, addition of purified GbpA protein did not affect *V. cholerae* aggregate formation, including in a Δ*gbpA* mutant. (Fig. S10).

The role of the LapX C-terminal domains and LapX-driven catalysis in *V. cholerae* aggregate formation was examined by, respectively, the addition of purified LapX ΔC and purified LapX FL S319A to the aggregation timing assay. The LapX C-terminal domain is dispensable for aggregation formation as the LapX ΔC protein led to the same aggregation timing as purified LapX FL in the *V. cholerae* Δ*lapX* mutant (Fig. 5*D*). LapX containing the catalytically inactive S319A mutation, when present in either LapX FL or LapX ΔC, failed to rescue the aggregation timing defect displayed by the Δ*lapX* strain (Fig. 5*D*). These data exactly parallel the *in vitro* findings that LapX proteolytic activity, but not the LapX C-terminal domain, is required to process the Lap protease and validate why both LapX and Lap are required for WT aggregation timing in *V. cholerae*.

## Discussion

LapX and Lap are secreted proteases that have recently been shown to play roles in the liquid-based aggregation process of *V. cholerae*. Here we show that LapX undergoes autoprocessing in the presence of calcium and then facilitates the maturation of Lap. While LapX is a member of a serine protease subfamily with no other characterized homologs, it contains a conserved catalytic triad of these serine proteases and exhibits proteolytic activity. The *lapX* gene resides adjacent to *lap* or to a gene encoding a serine protease in many *Vibrio* genomes suggesting that this pathway may be widespread in *Vibrio* species. Secreted proteases like LapX and Lap are often produced as zymogens (39), which presumably provides control over their temporal and spatial activities. As observed here, zymogen processing often involves an N- or C-terminal inhibitory prodomain that occludes the active site cleft. Our findings are consistent with known bacterial proteolytic cascades (40). While both proteases were expressed successfully as zymogens, over time, they underwent stochastic maturation during purification and/or storage making pure zymogen difficult to consistently obtain. Maturation occurred either through autoprocessing (in the case of LapX) or possibly due to an unknown *E. coli* protease since protease inhibitors were not used during purification to avoid complications with downstream assays.

LapX has a substrate preference that is primarily determined by Glu and Gln at the P1 site. Nonetheless, LapX demonstrated relatively low activity on peptide substrates containing these amino acids. Low activity proteases are not uncommon, with LapX exhibiting modestly lower activity than that reported for KSHV Pr (41), another serine protease. Compared to commonly studied serine proteases, KSHV Pr also has low activity. That feature is attributed to its Ser-His-His catalytic triad configuration rather than the canonical Ser-His-Asp triad. Given that LapX has a Ser-His-Asp triad, its slow rate may be due to a yet unidentified factor important for full activity, perhaps one that interacts similarly to PDZ domains for DegS (42) and DegP (43). Since our internally quenched substrates contain only eight residues, it is also possible that these substrates do not engage exosites that enhance the degradation of folded proteins or longer peptides (44). This lack of engagement would also explain why cleavage with a peptide derived from the C-terminal region of the M28 domain of Lap exhibits no activity when treated under the same conditions as the N-terminal peptide.

LapX is distinct from most other serine proteases, as AEBSF appears to inactivate the enzyme only at high concentrations. We also do not fully understand the LapX requirement for Ca^2+^; however, our finding is consistent with *V. cholerae* aggregation depending on LapX (and Lap) and occurring only in the presence of Ca^2+^. One possibility is that an autodegradation switch occurs in the presence of Ca^2+^ as some proteases undergo self-degradation (45) after completing their biological functions and thus Ca^2+^ may play a role coordinating the aggregation program.

Lap was confirmed to be a leucyl aminopeptidase that prefers hydrophobic and threonine residues, most notably methionine (norleucine in MSP-MS) at the cleavage site. This observation is consistent with *Vp*AP activity, where cleavage of methionyl- and norleucyl-substrates occurs more rapidly than for leucyl-substrates (28). Our analyses of Lap ΔPPC and Lap I103-Q396 show that Lap requires its N-terminal prosegment for proper folding when expressed and transported to the *E. coli* periplasm, consistent with other members of this enzyme family. The fully active protein binds two Zn^2+^ atoms. The C-terminal prepeptidase domain does not affect Lap substrate preference but is partially inhibitory, as its removal causes a four-fold increase in catalytic efficiency (*k*_cat_/*K*_*M*_) on a leucine aminopeptidase substrate. Lap activity is further enhanced by treatment with LapX, which yields an 18-fold increase in catalytic efficiency, suggesting that the Lap N-terminal prepeptidase domain is also partially inhibitory. These results agree with characterization of the N-terminal and C-terminal domains of the *Vp*AP homolog (46).

**Figure 6:**
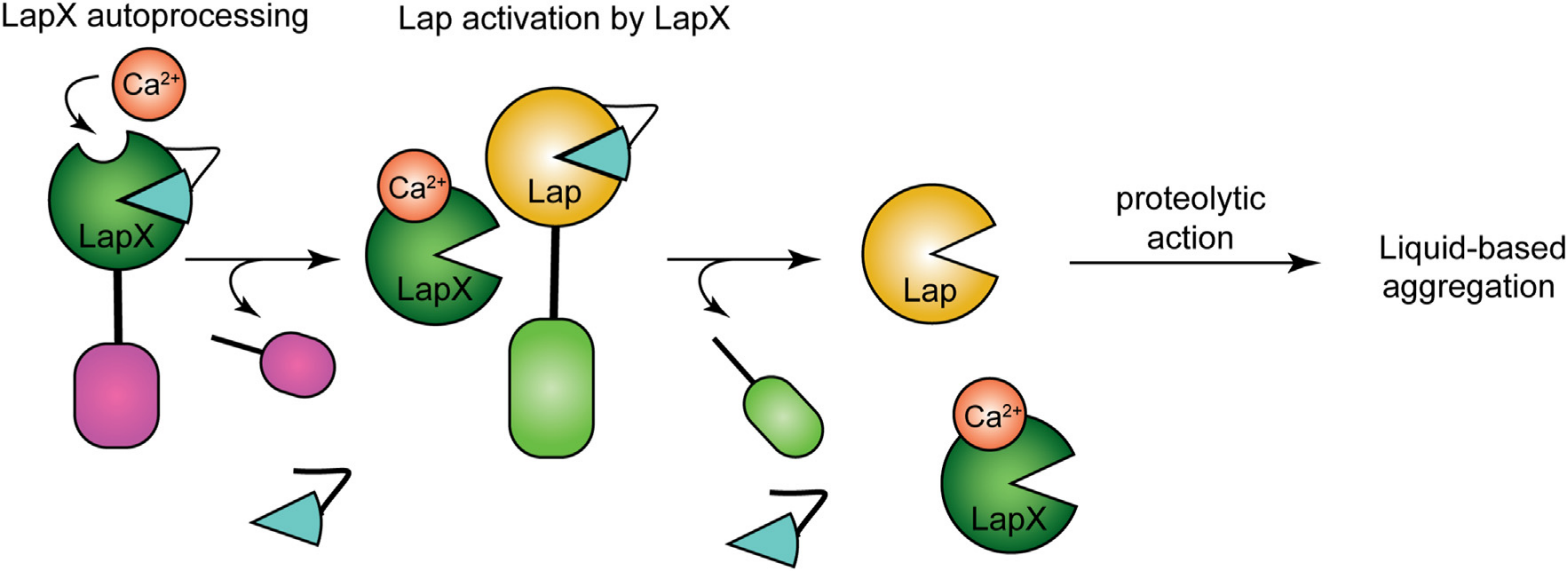
**Model for LapX and Lap roles in V. cholerae aggregation.** In the presence of calcium, LapX is autoprocessed (*i.e.*, LapX loses the cyan-colored prosegment represented by the pie-slice shape and the pink C-terminal domain) to its most active form (dark green pacman). Next, LapX cleaves the propeptide from Lap enabling Lap protease maturation (gold pacman) (LapX removes the Lap prosegment region represented by the cyan pie-slice shape and light green PPC domain). Subsequently, Lap processes an unknown substrate that leads directly or indirectly to V. cholerae aggregation.

When LapX and Lap are exogenously added back to *V. cholerae* Δ*lapX* and Δ*lap* mutants, respectively, they restore the defects in aggregation timing. The addition of the proteases at a 1:1 ratio rescued WT aggregation timing. *V. cholerae* aggregation occurs in late stationary phase so only one cell density, that mimics stationary phase, was examined in the protein supplementation assays (15). Aggregation does not appear to require a DNA scaffold as previous experiments show that addition of DNase does not affect aggregation. A model for how these enzymes act in this pathway is presented in Figure 6. LapX undergoes autoprocessing, then it processes Lap, which can then act on a substrate that is required for aggregation, such as that described in other auto-aggregation pathways (12). Alternatively, the proteases may act by degrading a repressor of aggregation. LapX and Lap do not appear to fulfill redundant functions, as the addition of Lap to a *lapX* deletion mutant cannot restore proper aggregation timing and vice versa. A mutant harboring a quadruple deletion of *lap*, *lapX*, *hapA*, and *prtV* exhibits a more extreme aggregation timing defect than the *lap*, *lapX* double mutant (15), indicating that these other proteases could play redundant roles with respect to the Lap and LapX pair. HapA and PrtV may also facilitate maturation of Lap and LapX. Defining the functions of HapA and PrtV in *V. cholerae* aggregation is a focus of ongoing studies. The LapX C-terminal domain is likely not the aggregation initiator, as aggregation rescue occurs following addition of LapX ΔC. This result, coupled with the findings that the LapX FL S319A and LapX ΔC S319A proteins cannot rescue aggregation, strongly supports the conclusion that LapX-mediated proteolytic action is what is required for aggregation, and the target of LapX action is another extracellular protein, presumably Lap.

Overall, these results provide the molecular underpinnings for a unique mechanism of control of bacterial aggregation as other proteases purportedly important for aggregation drive escape from aggregation rather than initiation of aggregation (13, 14). While physiological substrate identification has been hindered by the highly insoluble nature of the *V. cholerae* aggregates, future studies using the substrate preference data uncovered here combined with proteomic experiments could clarify the overall process and add important mechanistic insight.

## Experimental procedures

### Generation of constructs

Genomic DNA was isolated from *V. cholerae* O395 using the Invitrogen TRIzol DNA extraction kit (Thermo Fisher Scientific) following the manufacturer's protocol. *vca0811*, encoding GbpA (UniprotKB ID: A6XA54), was cloned into pET46. *vca0812*, encoding LapX (Uniprot KB ID: Q9KLD4; MEROPS MER0216699) and *vca0813* encoding Lap (UniprotKB ID: Q9KLD3; MEROPS MER1153747) were cloned into pET22b by first removing the DNA specifying the native signal peptides and replacing that with the DNA encoding the pelB leader peptide. The constructs also harbored C-terminal poly-histidine tags. All cloning primers are reported in Table S1. Cloning was performed using the Gibson assembly mix from NEB according to the manufacturer’s protocol. Plasmids were transformed into *E*. *coli* XL1 Blu cells (QB3 Macrolab) and grown on LB agar plates containing 100 μg/mL ampicillin (Sigma). Single colonies were isolated and grown overnight at 37 °C. DNA was harvested using Zymo Research miniprep kits according to manufacturer’s protocol. Constructs were verified by sequencing and then used for protein production. Q5 kits (NEB) were used to generate the point mutation and truncations according to the manufacturer’s protocol.

### AlphaFold2 and AlphaFill

Sequences of *vca0813* and *vca0812* were retrieved from Uniprot (47) and the signal peptide was trimmed according to SignalP 6.0 (48). The resulting sequence was input into the Google Colab notebook (https://colab.research.google.com/github/deepmind/alphafold/blob/main/notebooks/AlphaFold.ipynb) to perform the AlphaFold2 program for each peptide (30). The result for *vca0813* was used as an input for the AlphaFill prediction tool https://alphafill.eu/ (29). Structures were viewed using ChimeraX (49); the structure of *Vp*AP (PDB:1AMP) was overlayed against the AlphaFill prediction of Lap using the matchmaker tool.

### Purification of LapX and Lap

*E. coli* BL21 DE3 Ros2 pLysS cells (QB3 Macrolab) were transformed with the pET22b plasmid harboring a *lapX* or *lap* construct. All constructs contained a pelB leader sequence for periplasmic transport as well as a C-terminal poly-histidine tag for IMAC purification. Transformants containing plasmids with *lap* were grown in LB medium supplemented with 100 μM ZnSO_4_. In all cases, overnight cultures were grown for 16 h at 37 °C in LB medium supplemented with 100 μg/ml ampicillin and 25 μg/ml chloramphenicol. 1:80 dilutions of overnight cultures were made in TB medium with antibiotics and incubated at 37 °C until the cells reached an A_600_ of 0.6 to 0.8. Cultures were cooled to 18 °C, protein production was induced with 100 μM IPTG for 16 h, and the cells were harvested by centrifugation for 20 min at 4000 x *g*. Pellets were resuspended 1:4 (w/v) in 30% sucrose 50 mM Tris pH 8.0 0.1 mg/mL DNase and 0.1 mg/ml lysozyme. Protease inhibitors were not included in the purification to avoid interference with downstream assays. Samples were rotated for 30 min at 4 °C followed by centrifugation at 5000 x *g* for 55 min. The supernatant was retained, and the cells were resuspended 1:4 (w/v) in 5 mM MgCl_2_ 5 mM Tris pH 8.0, 0.1 mg/mL DNase, and 0.1 mg/mL lysozyme. This suspension was rotated for 30 min at 4 °C followed by centrifugation at 8000 x *g* for 10 min. Further clarification of the supernatants was performed by centrifugation at 3300 x *g* for 1 h when necessary. Supernatants from the two treatments were combined and subjected to a His60 Ni-IMAC column equilibrated with buffer B (50 mM Hepes (RPI), 300 mM NaCl, 5% glycerol, pH 8.0, and 10 mM imidazole) or the same resin charged with Zn^2+^ for all Lap constructs, as purification of Lap using Ni-IMAC led to Ni contamination. Columns were washed with 20 CV of buffer B and the proteins were eluted in 10 CV fractions in a stepwise manner using buffer B with increasing concentrations of imidazole (10 mM, 40 mM, 80 mM, 120 mM, 250 mM). The fractions that were judged pure by SDS-PAGE were combined and concentrated and the buffer was exchanged using PD-10 (GE Healthcare) according to the manufacturer’s protocol into 50 mM Hepes pH 7.6, 5% glycerol. Samples were stored at −80 °C prior to use.

### Purification of GbpA

GbpA was produced in *E. coli* DE3^∗^ BL21 cells (QB3 Macrolab) that were grown in LB (RPI) supplemented with 100 μg/mL of ampicillin for 16 h at 18 °C. Flasks containing TB (RPI) with 100 μg/mL ampicillin were inoculated using a 1:80 dilution of the overnight culture. Once the culture reached an optical density (A_600_) of 0.8 to 1, the cells were cooled to 18 °C, protein production was induced with 100 μM IPTG for 16 h, and the cells were collected by centrifugation at 4000 x *g* for 20 min at 4 °C. The pellets were resuspended 1:4 (w/v) in ice-cold 50 mM Na_2_HPO_4_, 300 mM NaCl, 10 mM imidazole (Oakwood Chemical), 5% glycerol, pH 8 containing 1 mM AEBSF (RPI), 1 mM benzamidine (RPI), 0.1 mg/mL DNase (Sigma-Aldrich), and 0.1 mg/mL lysozyme (Thermo Fisher Scientific). The cells were lysed by homogenization. The lysate was clarified by centrifugation at 40,000 rpm for 40 min at 4° C. The clarified supernatant was passed through buffer A (50 mM Na_2_HPO_4_, 300 mM NaCl, 5% glycerol, pH 8.0, 10 mM imidazole) on an equilibrated His60 Ni-IMAC column (Takara Bio) and eluted stepwise using buffer A with increasing concentrations of imidazole: 10 mM, 40 mM, 80 mM, 120 mM, 250 mM. Fractions containing pure protein were determined by SDS-PAGE, then pooled and concentrated with 10 kDa VivaSpin concentrators (Sartorius). The tagged GbpA was treated with Enterokinase light chain (NEB) following the manufacturer’s protocol, except CaCl_2_ (Amresco) was limited in the reaction to 500 μM as higher concentrations caused precipitation. The completed reaction was subjected to His60 Ni-IMAC chromatography (Takara Bio) and washed with buffer A containing 10 mM imidazole. Both the flow-through and wash fractions were combined at this stage. In addition to GbpA, there was a ∼38 kDa species previously reported to be a degradation product of the carbohydrate-binding module domain (20). The GbpA protein was transferred to SnakeSkin Dialysis Tubing (Thermo Fisher Scinetific) and dialyzed against a reconstitution buffer containing 50 mM NaOAc, 50 mM NaCl, 5 μM CuSO_4_, pH 5.5 overnight at 4 °C. GbpA was next dialyzed against a buffer of 50 mM MOPS (EMD Millipore), 50 mM NaCl, pH 7.0 overnight at 4 °C to remove unbound copper, concentrated, and stored at 4 °C prior to use. Attempts to remove the ∼38 kDa species using an S75 sizing column were unsuccessful. Confirmation of 1:1 copper loading of the GbpA was carried out by equilibrating 100 μM GbpA protein with 10 mM bathocuproine disulfonate (Sigma) and 1 mM ascorbic acid in a buffer of 50 mM MOPS, 50 mM NaCl, pH 7 for 1 h at RT. Copper absorption was measured at 483 nm and compared to a standard curve to determine the copper concentration in the enzyme.

### Inductively coupled plasma-mass spectrometry

Lap and LapX samples were digested at RT in 2% HNO_3_ in 15-mL conical tubes (Sarstedt). These preparations were subjected to analysis on an Agilent 8800 triple quadrupole inductively coupled plasma-mass spectrometry instrument. Internal standards Sc, Y, and Re were added to samples inline. The concentration of each metal was determined by interpolating with a standard curve.

### Edman sequencing

Samples containing 10 μM LapX and 10 μM Lap were incubated for 10 min in 50 mM TAPS (Thermo Fisher Scientific) pH 9.0. An aliquot of that reaction (10 μL) was quenched by adding 5 μL of 6× SDS loading buffer. Reactions were subjected to SDS-PAGE analysis along with untreated 10 μM LapX and 10 μM Lap controls and transferred to a PVDF membrane (G-Biosciences). The membrane was stained with Coomassie Brilliant Blue and wrapped in plastic wrap to prevent contamination. The membrane was sent to UC Davis for sequencing. The first five amino acids in each band of the mixed reaction were analyzed and compared to amino acid standards. There were at least three main peptides in the Lap mixture, so the first nine possibilities (three peptides x three amino acid positions) were searched in the Lap peptide sequence. Only one returned with a possible peptide from Lap (IPAPS). A four amino acid peptide was matched to LapX (GINE). Other combinations were not present in either protein sequence.

### Electrospray ionization time of flight liquid chromatography mass spectrometry

Water was purified to a resistivity of 18.2 MΩ cm (at 25 °C) using a Milli-Q Gradient ultrapure water purification system (Millipore). Acetonitrile (Optima grade, 99.9%, Thermo Fisher Scientific), formic acid (1 mL ampules, 99+%, Pierce), and the purified water were used to prepare mobile phase solvents S3 for LC-MS. Electrospray ionization mass spectrometry of protein samples was performed using an Agilent 1260 series liquid chromatograph outfitted with an Agilent 6224 time-of-flight LCMS system. The LC was equipped with a Proswift RP-4H (monolithic phenyl, 1.0 mm × 50 mm, Dionex) analytical column. Solvent A was 99.9% water/0.1% formic acid (v/v) and solvent B was 99.9% acetonitrile/0.1% formic acid (v/v). Proteins were buffer exchanged into 25 mM ammonium bicarbonate pH 7.5 using Bio-Spin 6 (Bio-Rad) columns according to the manufacturer’s protocol and then subjected to centrifugation through 0.22 μm cellulose acetate centrifugal spin filters. Samples of 1 to 5 μL were injected, corresponding to 10 pmol of protein, onto the column. Following sample injection, a 5 to 100% solvent B elution gradient was applied at a 0.30 mL/min flow rate over 8 min. Data were collected and analyzed by deconvolution of the entire elution profile (using Agilent Mass Hunter Qualitative Analysis B.05.00) to provide reconstructed mass spectra representing the entire sample. Spectra were analyzed with the open-source software Chartograph (www.chartograph.com).

### SDS-PAGE protein processing assays

For LapX autoprocessing, 10 μM LapX was added to a buffer that contained buffer C (1 mM CaCl_2_ and 20 mM TAPS pH 8.0) and incubated at RT. At each time point, 10 μL was quenched with 5 μL of 6× SDS loading buffer. For LapX processing of Lap, 1 μM of Lap FL or Lap ΔPPC was treated with 10 nM of LapX FL in buffer C. For inhibition testing, Roche Complete EDTA Free tablets (Roche) were prepared as directed, and AEBSF (final concentration of 1 mM) or benzamidine (final concentration 40 mM) were first dissolved in MilliQ H_2_O. Inhibitors were preincubated with LapX ΔC for 30 min, and experiments were initiated by addition of Lap FL. Ten micromolars of LapX ΔC was incubated with 10 μM of ZnCl_2_, 1 mM MgCl_2_, or 1 mM CaCl_2_ to test for other activating cofactors in 20 mM TAPS pH 8.0. All gels were imaged using the Bio-Rad Stain-Free technology.

### SSN generation and genetic alignment

Multiple sequence alignments were performed using the Clustal Omega tool (https://www.ebi.ac.uk/Tools/msa/clustalo/) and visualized using the box shade ExPASy tool (source code https://sourceforge.net/projects/boxshade/) using the RTF new option with default options. SSNs were generated using the EFI-EST website through the BLAST function (50) with a limit of 10,000 sequences. An alignment score of 130 was used for the LapX SSN to isolate the sequences that were from organisms in the *Vibrio* genus and to perform a genome neighborhood analysis on related organisms. A genome neighborhood diagram was created using the SSN as an input with default settings. Even at a high alignment score, Lap could not be separated into a cluster that was distinct by genus.

### *p*-Nitroanilide assay for Lap kinetics

The cleavage of *p*-nitroanilides to yield *p*-nitroaniline was used to assay the activity of Lap FL and Lap ΔPPC. Leucine *p*-nitroanilide (RPI), valine *p*-nitroanilide, and isoleucine *p*-nitroanilide (Santa Cruz Biotechnology) were tested. Absorbance at 405 nm was measured to quantify the release of *p*-nitroaniline over time. Reactions were carried out at RT in triplicate in 96-well plates (Corning). Measurements were made in a SpectraMax Pro 5 instrument (Molecular Devices), and data were exported from the software SoftMax Pro 5.4. Contents of wells were mixed for 5 s prior to data collection. The extinction coefficient of *p*-nitroaniline (Sigma) in 20 mM TAPS at pH 8.0 was determined by generating a standard curve for the absorbance at 405 nm using SpectraMax Pro 5. Initial rates were calculated from the slopes of the linear ranges using the extinction coefficients determined by the standard curves. To assess the activation of Lap FL by LapX FL, Lap FL and Lap ΔPPC (1 μM) were incubated with CaCl_2_·2H_2_O (1 mM) and LapX FL (10 nM) at RT at pH 8.0 for 10 min. For substrate kinetics, the *p*-nitroanilide assay was carried out at multiple concentrations of leucine *p*-nitroanilide. Data from control wells lacking the substrate were measured and used as the baselines to subtract from the output from wells with substrate prior to linear regression. The rate of product formation *versus* substrate concentration was plotted and fitted to the *k*_*cat*_ equation using GraphPad Prism 9.4.1 to solve for *k*_*cat*_ and *K_M_*.

### Determination of the native LapX cleavage site

To assess LapX activity on a physiological substrate, two peptides that correspond to the N- and C- terminal regions of the Lap M28 protease domain, MPESIANFTIPAPSQQD and SSQVPDDSVLQDGTAKIN, respectively, were obtained (GenScript). Ten micromolar of test peptide was incubated at RT for 1 h in the presence of 500 nM LapX FL in 50 mM TAPS pH 8.0 with 1 mM CaCl_2_. The reaction was quenched by boiling for 5 min at 80 °C, and the sample was subjected to centrifugation to remove precipitated protein. Two microliters of the resulting supernatant was injected onto an Acclaim Polar Advantage II, C18, 3 μm, 120 Å, 2.1 × 150 mm column (Thermo Fisher Scientific) equipped on an Agilent 1260 infinity II LC system attached to an Agilent QTOF model 6530 MS. Peptides were eluted at 300 nL/min on a gradient with two solvents. Solvent A (LC-MS grade H_2_O with 0.1% formic acid) and solvent B (acetonitrile with 0.1 % formic acid) were used in the elution as follows: 95 % A for 1.5 min then a linear gradient to 55% A for 6.5 min, followed by a linear gradient to 5 % A over 7 min, and then the solvent was held constant. The column was re-equilibrated to 95% A for 7 min.

### Peptide substrate generation

An internally quenched fluorogenic peptide was made containing the sequence corresponding to the P1-4 and P1′-P4′ sites (MMPESIAN) of the Lap-based peptide on which LapX exhibited activity. The fluorophore, MCA, was conjugated to N_ε_ of a Lys residue at the N-terminus, and DNP was conjugated to N_ε_ of a Lys residue at the C-terminus. Similarly, internally quenched peptide substrates were designed with the eight amino acids according to the peptide corresponding to the highest observed cleavage as determined by ion count from MSP-MS experiments. The two peptides selected were VTAESLMS and MRQQYLNT with Glu and Gln in the P1 positions, respectively. These two peptides showed the highest cleavage (measured by spectral counts) for each P1 position. Peptides of the sequence NH_2_-K(MCA)MMPESIANK(DNP)-COOH, NH_2_-K(MCA)VTAESLMSK(DNP)-COOH, NH_2_-K(MCA)MRQQYLNTK(DNP)-COOH were synthesized and purified following a procedure published previously (51). Dried solids were stored at −20 °C until use. Powders were reconstituted to a final concentration of 200 mM in DMSO. The 200 mM stock and subsequent dilutions could be stored at −20 °C for up to 2 weeks with no degradation observed.

### Fluorescent internally quenched peptide kinetics

Fresh protein from the −80 °C freezer was diluted to 1 μM in 50 mM TAPS pH 8.0 and samples were transferred to wells of a prewarmed Corning 384 well black polystyrene plate. Reactions were initiated by the addition of an equal volume of 2× substrate (diluted from a DMSO stock of 200 mM peptide into TAPS pH 8.0, 2 mM CaCl_2_). The resultant reaction mixture contained 1.25% DMSO and 1 mM CaCl_2_. Each well was mixed gently by pipetting once and then absorbance was measured on a Biotek Synergy Neo 2 Plate reader. The plate was shaken for 10 s, and the increase in emission signal at 420 nm when excited with 360 nm wavelength light was monitored at 37 °C for 30 min. The initial slopes were fit to a standard curve of known MCA (Sigma) to determine rates. These data were plotted and analyzed using GraphPad Prism 9.2.0 using the *k*_*cat*_ fitting equation to generate Michaelis–Menten kinetic parameters. The dose-response curve of AEBSF was generated in a similar manner. In this case, the protein was first incubated with 2× final concentration of inhibitor at RT for 1 h, warmed to 37 °C for 2 min, and the reaction initiated by the addition of an equal volume of 100 μM substrate. Obtained slopes were normalized to the wells that contained no inhibitor. For inhibition by EDTA, because the buffer contained 1 mM CaCl_2_, samples were incubated with 2 mM EDTA for 1 h at 25 °C and warmed to 37 °C for 2 min. Reactions were then initiated with an equal volume of 100 μM substrate. IC_50_ measurements were not performed as the buffer contained a divalent cation that would complicate interpretation.

### Multiplex substrate profiling-mass spectrometry

The 228-peptide library reported previously (32, 51) was incubated at a final concentration of 1 μM with 50 nM of each protease construct. Timepoints were taken at 15 min, 1 h, and 4 h, followed by immediate quenching with an equal volume of 6 M Guanidium HCl. Samples were acidified by the addition of 20% formic acid to a final concentration of 1.25%. The quenched samples were desalted using Millipore Ziptips, which were first activated by aspirating 15 μL of 50% acetonitrile 0.2% formic acid in LC-MS grade water 3 times and discarding the flowthrough (FT). The tips were next washed by aspirating 15 μL of 0.2% formic acid in LC-MS grade water 3 times and the FT was discarded. Samples were bound by aspirating 15 μL 10 times in the tips. The tips were washed with 15 μl of 0.2% formic acid in LC-MS grade water 5 times and the FT was discarded. Lastly, the bound peptides were eluted by aspirating 15 μl of 50% acetonitrile 0.2% formic Acid in LC-MS grade water 5 times with transfer to a nonstick 0.5 ml Axygen maximum recovery tube (Corning). Samples were dried using a Gene Vac EZ bio speed vacuum. Samples were resuspended in 0.1% formic acid in LC-MS grade water. Two microliters of each sample was injected onto a PepMAP RSLC C18 column 3 μM 100A, 75 μM x 15 cm (Thermo Fisher Scientific) on a 10,000-psi nanoACQUITY Ultra Performance Liquid Chromatography System (Waters) followed by a Q Exactive Plus Hybrid Quadrupole-Orbitrap (Thermo Fisher Scientific). Peptides were eluted at a flow rate of 400 nL/min using a 90 min gradient of two buffers A (0.1% formic acid in LCMS-grade water) and B (0.1% formic acid in acetonitrile). The linear gradients were as follows: 2% B for 15 min, to 25% B over 43 min, 37% B over 6 min, 40% B over 3 min, to 80% B over 3 min, to 2% B over 2 min, followed by re-equilibration at 2% B for 13 min. The resulting spectra were extracted using the Pava method as previously described (32, 51). The program Prospector was used to identify cleavages against a database containing the full set of all possible cleavages in the library. Peptide cleavages that were observed in a no-enzyme control sample were subtracted from the dataset. Observed cleavages for each timepoint were combined and analyzed using ICE-Logo (https://iomics.ugent.be/icelogoserver/) accessed 06/04/22 and the frequency plot generated *versus* a reference set containing all possible peptide cleavages. The top peptides that contained (E) or (Q) in the P1 position over all six replicates (LapX FL and LapX ΔPPC) were selected for kinetic studies.

### *V. cholerae* aggregation assay

The strains used in this study are listed in Table S2. *V. cholerae* aggregation assays were performed as previously described (15). Briefly, *V. cholerae* strains were grown in tubes in the outer ring of a rotor drum (New Brunswick) at 30 °C in LB (Fisher Bioreagents). LapX FL, LapX ΔC, LapX FL S319A, LapX ΔC S319A, Lap FL, and/or GbpA were added at 100 nM at the time of inoculation, which we call T = 0. Starting at T = 18 h, and continuing hourly until aggregates formed, 100 μL of each sample was moved to a 96-well microtiter dish (MatTek) for fluorescence visualization. Aggregates were imaged with a 10× objective on a scanning confocal microscope (Leica) as described previously (4). Each experiment was performed in biological triplicate on two separate days.

## Data availability

Data not presented in the article is available upon request.

## Supporting information

This article contains supporting information (32).

## Conflict of interest

The authors declare that they have no conflicts of interest with the contents of this article.

## References

[1] Vestby L.K., Grønseth T., Simm R., Nesse L.L. (2020). Bacterial biofilm and its role in the pathogenesis of disease. Antibiotics.

[2] Høiby N. (2017). A short history of microbial biofilms and biofilm infections. APMIS.

[3] Trunk T., Khalil H.S., Leo J.C. (2018). Bacterial autoaggregation. AIMS Microbiol..

[4] Jemielita M., Wingreen N.S., Bassler B.L. (2018). Quorum sensing controls Vibrio cholerae multicellular aggregate formation. Elife.

[5] Boyaci H., Shah T., Hurley A., Kokona B., Li Z., Ventocilla C. (2016). Structure, regulation, and inhibition of the quorum-sensing signal integrator LuxO. PLOS Biol..

[6] Papenfort K., Silpe J.E., Schramma K.R., Cong J.-P., Seyedsayamdost M.R., Bassler B.L. (2017). A Vibrio cholerae autoinducer-receptor pair that controls biofilm formation. Nat. Chem. Biol..

[7] Faruque S.M., Biswas K., Udden S.M.N., Ahmad Q.S., Sack D.A., Nair G.B. (2006). Transmissibility of cholera: in vivo-formed biofilms and their relationship to infectivity and persistence in the environment. Proc. Natl. Acad. Sci. U. S. A..

[8] Tamayo R., Patimalla B., Camilli A. (2010). Growth in a biofilm induces a hyperinfectious phenotype in Vibrio cholerae. Infect. Immun..

[9] Nelson E.J., Chowdhury A., Harris J.B., Begum Y.A., Chowdhury F., Khan A.I. (2007). Complexity of rice-water stool from patients with Vibrio cholerae plays a role in the transmission of infectious diarrhea. Proc. Natl. Acad. Sci. U. S. A..

[10] Khalil H.S., Øgaard J., Leo J.C. (2020). Coaggregation properties of trimeric autotransporter adhesins. Microbiologyopen.

[11] Béchon N., Jiménez-Fernández A., Witwinowski J., Bierque E., Taib N., Cokelaer T. (2020). Autotransporters drive biofilm formation and autoaggregation in the diderm firmicute veillonella parvula. J. Bacteriol..

[12] Nwoko E.Q.A., Okeke I.N. (2021). Bacteria autoaggregation: how and why bacteria stick together. Biochem. Soc. Trans..

[13] Hendrixson D.R., Geme J.W.S. (1998). The Haemophilus influenzae Hap serine protease promotes adherence and microcolony formation, potentiated by a soluble host protein. Mol. Cell.

[14] Habouria H., Pokharel P., Maris S., Garénaux A., Bessaiah H., Houle S. (2019). Three new serine-protease autotransporters of Enterobacteriaceae (SPATEs) from extra-intestinal pathogenic Escherichia coli and combined role of SPATEs for cytotoxicity and colonization of the mouse kidney. Virulence.

[15] Jemielita M., Mashruwala A.A., Valastyan J.S., Wingreen N., Bassler B.L. (2021). Secreted proteases control the timing of aggregative community formation *in vibrio* cholerae. mBio.

[16] Finkelstein R.A., Boesman-Finkelstein M., Holt P. (1983). Vibrio cholerae hemagglutinin/lectin/protease hydrolyzes fibronectin and ovomucin: F. M. Burnet revisited. Proc. Natl. Acad. Sci. U. S. A..

[17] Vaitkevicius K., Rompikuntal P.K., Lindmark B., Vaitkevicius R., Song T., Wai S.N. (2008). The metalloprotease PrtV from Vibrio cholerae Purification and properties. FEBS J..

[18] Jude B.A., Martinez R.M., Skorupski K., Taylor R.K. (2009). Levels of the secreted Vibrio cholerae attachment factor GbpA are modulated by quorum-sensing-induced proteolysis. J. Bacteriol..

[19] Bhowmick R., Ghosal A., Das B., Koley H., Saha D.R., Ganguly S. (2008). Intestinal adherence of Vibrio cholerae involves a coordinated interaction between colonization factor GbpA and mucin. Infect. Immun..

[20] Wong E., Vaaje-Kolstad G., Ghosh A., Hurtado-Guerrero R., Konarev P.V., Ibrahim A.F.M. (2012). The Vibrio cholerae colonization factor GbpA possesses a modular structure that governs binding to different host surfaces. PLoS Pathog..

[21] Van Heeke G., Denslow S., Watkins J.R., Wilson K.J., Wagner F.W. (1992). Cloning and nucleotide sequence of the Vibrio proteolyticus aminopeptidase gene. Biochim. Biophys. Acta Gene Struct. Expr..

[22] Stamper C.C., Bienvenue D.L., Bennett B., Ringe D., Petsko G.A., Holz R.C. (2004). Spectroscopic and X-ray crystallographic characterization of bestatin bound to the aminopeptidase from Aeromonas (Vibrio) proteolytica. Biochemistry.

[23] Holz R.C. (2002). The aminopeptidase from Aeromonas proteolytica: structure and mechanism of co-catalytic metal centers involved in peptide hydrolysis. Coord. Chem. Rev..

[24] Bzymek K.P., Swierczek S.I., Bennett B., Holz R.C. (2005). Spectroscopic and thermodynamic characterization of the E151D and E151A altered leucine aminopeptidases from Aeromonas proteolytica. Inorg. Chem..

[25] Guenet C., Lepage P., Harris B.A. (1992). Isolation of the leucine aminopeptidase gene from Aeromonas proteolytica. Evidence for an enzyme precursor. J. Biol. Chem..

[26] Toma C., Honma Y. (1996). Cloning and genetic analysis of the Vibrio cholerae aminopeptidase gene. Infect. Immun..

[27] Hartley M., Bennett B. (2009). Heterologous expression and purification of Vibrio proteolyticus (Aeromonas proteolytica) aminopeptidase: a rapid protocol. Protein Expr. Purif..

[28] Wagner F.W., Wilkes S.H., Prescott J.M. (1972). Specificity of Aeromonas aminopeptidase toward amino acid amides and dipeptides. J. Biol. Chem..

[29] Hekkelman M.L., de Vries I., Joosten R.P., Perrakis A. (2023). AlphaFill: enriching AlphaFold models with ligands and cofactors. Nat. Methods.

[30] Jumper J., Evans R., Pritzel A., Green T., Figurnov M., Ronneberger O. (2021). Highly accurate protein structure prediction with AlphaFold. Nature.

[31] Pandey K.C., Sijwali P.S., Singh A., Na B.-K., Rosenthal P.J. (2004). Independent intramolecular mediators of folding, activity, and inhibition for the plasmodium falciparum cysteine protease falcipain-2. J. Biol. Chem..

[32] O’Donoghue A.J., Eroy-Reveles A.A., Knudsen G.M., Ingram J., Zhou M., Statnekov J.B. (2012). Global identification of peptidase specificity by multiplex substrate profiling. Nat. Methods.

[33] Rohweder P.J., Jiang Z., Hurysz B.M., O’Donoghue A.J., Craik C.S. (2023). Multiplex substrate profiling by mass spectrometry for proteases. Methods Enzymol..

[34] Nyborg J.K., Peersen O.B. (2004). That zincing feeling: the effects of EDTA on the behaviour of zinc-binding transcriptional regulators. Biochem. J..

[35] Ivry S.L., Meyer N.O., Winter M.B., Bohn M.F., Knudsen G.M., O’Donoghue A.J. (2018). Global substrate specificity profiling of post-translational modifying enzymes. Protein Sci..

[36] Shannon D.A., Gu C., McLaughlin C.J., Kaiser M., van der Hoorn R.A.L., Weerapana E. (2012). Sulfonyl fluoride analogues as activity-based probes for serine proteases. ChemBioChem.

[37] Rendón-Ramírez A., Shukla M., Oda M., Chakraborty S., Minda R., Dandekar A.M. (2013). A computational module assembled from different protease family motifs identifies PI PLC from Bacillus cereus as a putative prolyl peptidase with a serine protease scaffold. PLoS One.

[38] Higaki J.N., Evnin L.B., Craik C.S. (1989). Introduction of a cysteine protease active site into trypsin. Biochemistry.

[39] Khan A.R., James M.N. (1998). Molecular mechanisms for the conversion of zymogens to active proteolytic enzymes. Protein Sci..

[40] Konovalova A., Søgaard-Andersen L., Kroos L. (2014). Regulated proteolysis in bacterial development. FEMS Microbiol. Rev..

[41] Pray T.R., Reiling K.K., Demirjian B.G., Craik C.S. (2002). Conformational change coupling the dimerization and activation of KSHV protease. Biochemistry.

[42] Walsh N.P., Alba B.M., Bose B., Gross C.A., Sauer R.T. (2003). OMP peptide signals initiate the envelope-stress response by activating DegS protease *via* relief of inhibition mediated by its PDZ domain. Cell.

[43] Iwanczyk J., Damjanovic D., Kooistra J., Leong V., Jomaa A., Ghirlando R. (2007). Role of the PDZ domains in Escherichia coli DegP protein. J. Bacteriol..

[44] Jabaiah A.M., Getz J.A., Witkowski W.A., Hardy J.A., Daugherty P.S. (2012). Identification of protease exosite-interacting peptides that enhance substrate cleavage kinetics. Biol. Chem..

[45] López-Otín C., Bond J.S. (2008). Proteases: multifunctional enzymes in life and disease. J. Biol. Chem..

[46] Bzymek K.P., D’Souza V.M., Chen G., Campbell H., Mitchell A., Holz R.C. (2004). Function of the signal peptide and N- and C-terminal propeptides in the leucine aminopeptidase from Aeromonas proteolytica. Protein Expr. Purif..

[47] The UniProt Consortium (2023). UniProt: the universal protein knowledgebase in 2023. Nucleic Acids Res..

[48] Teufel F., Almagro Armenteros J.J., Johansen A.R., Gíslason .H., Pihl S.I., Tsirigos K.D. (2022). SignalP 6.0 predicts all five types of signal peptides using protein language models. Nat. Biotechnol..

[49] Pettersen E.F., Goddard T.D., Huang C.C., Meng E.C., Couch G.S., Croll T.I. (2021). UCSF ChimeraX: Structure visualization for researchers, educators, and developers. Protein Sci..

[50] Gerlt J.A., Bouvier J.T., Davidson D.B., Imker H.J., Sadkhin B., Slater D.R. (2015). Enzyme function initiative-enzyme similarity tool (EFI-EST): a web tool for generating protein sequence similarity networks. Biochim. Biophys. Acta Proteins Proteom..

[51] Zhao N., Bardine C., Lourenço A.L., Wang Y., Huang Y., Cleary S.J. (2021). *In Vivo* measurement of granzyme proteolysis from activated immune cells with PET. ACS Cent. Sci..

