# Supplementary material for: Proteases influence colony aggregation behavior in Vibrio cholerae
Source: J Biol Chem. 2023 Oct 26;299(12):105386. doi: 10.1016/j.jbc.2023.105386 (PMC10709122; doi:10.1016/j.jbc.2023.105386)
Supplement: Supporting information [file mmc1.docx]

# Supporting Information for

Proteases influence colony aggregation behavior in *Vibrio cholerae*

Tyler C. Detomasi^1,2*^, Allison E. Batka^1^, Julie S. Valastyan^3,4^, Molly A. Hydorn^1,5*^, Charles S. Craik^2^, Bonnie L. Bassler^3,4^, Michael A. Marletta^1,6,7†^.

1. Department of Chemistry, University of California, Berkeley, Berkeley, CA, 94720, United States
2. Department of Pharmaceutical Chemistry, University of California, San Francisco, San Francisco, CA, 94158, United States
3. Department of Molecular Biology, Princeton University, Princeton, NJ, 08544, United States
4. The Howard Hughes Medical Institute, Chevy Chase, MD, 20815, United States
5. Department of Microbiology and Immunology, College of Physicians and Surgeons, Columbia University, New York, NY, 10027, United States;
6. California Institute for Quantitative Biosciences, University of California, Berkeley, Berkeley, CA, 94720, United States
7. Department of Molecular and Cell Biology, University of California, Berkeley, Berkeley, CA, 94720, United States

*Current Affiliation

†Corresponding author

**Email:**  [marletta@berkeley.edu](mailto:marletta@berkeley.edu)


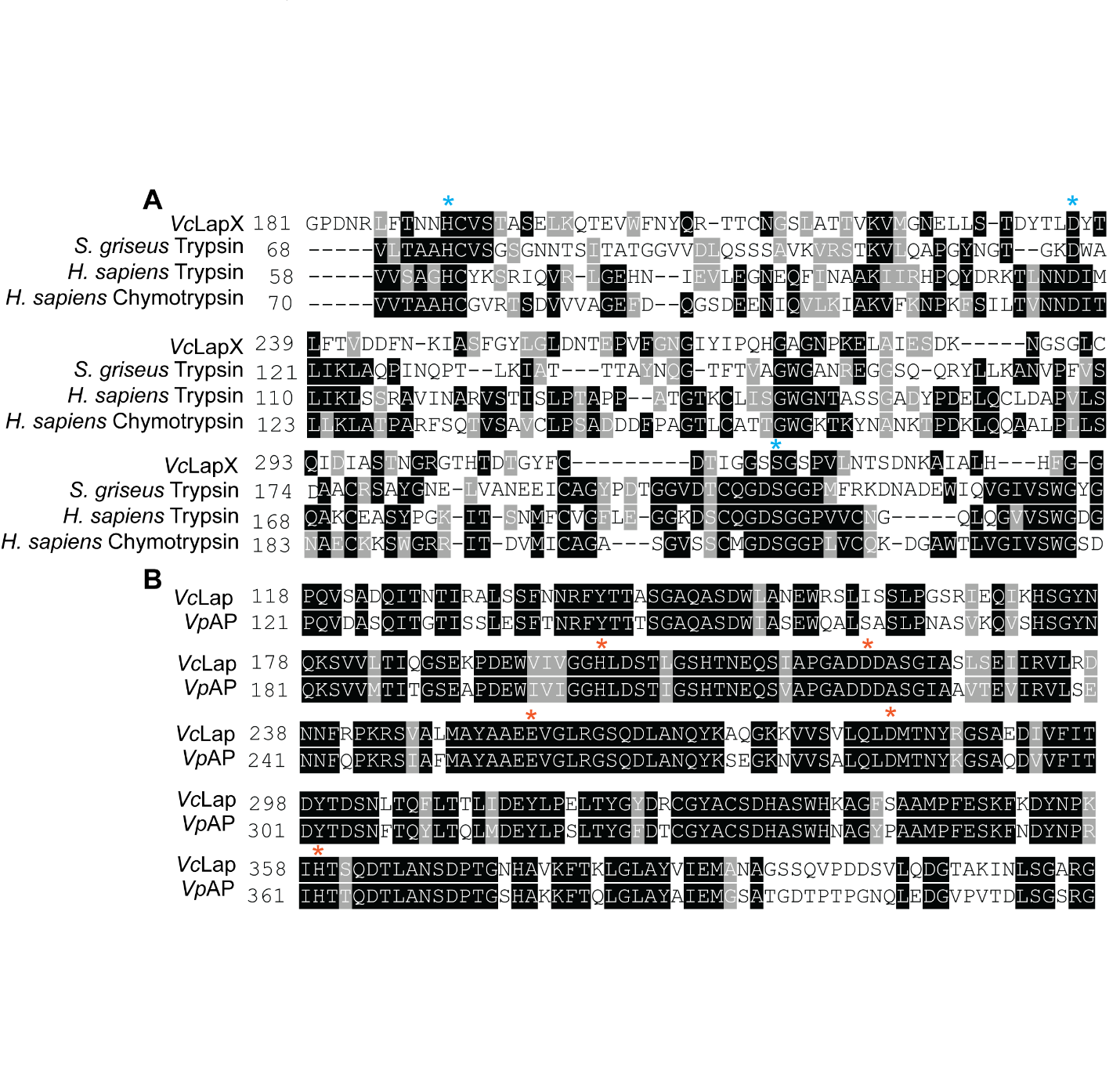


**Supplemental Figure 1. Alignments of the LapX and Lap protease domains and homologs**. A. Alignment of LapX with a bacterial trypsin, human trypsin, and human chymotrypsin. The catalytic triad is conserved and denoted by blue asterisks. B. Alignment of *Vp*AP (*Vp* denotes *Vibrio proteolyticus*) and *V. cholerae* Lap. The Zn^2+^ binding residues are conserved and denoted with orange asterisks.


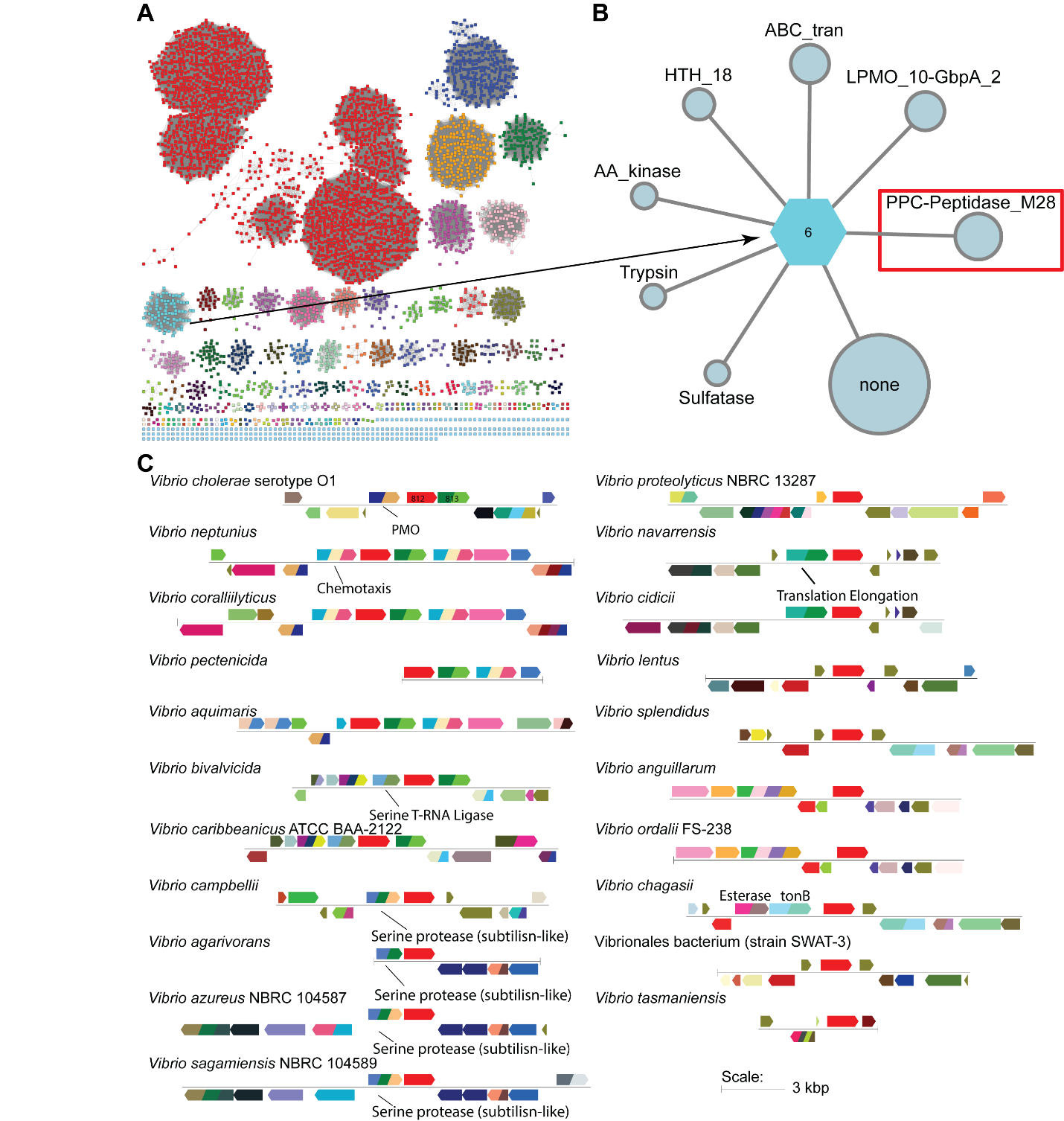


**Supplemental Figure 2. Sequence similarity network of proteases related to *V. cholerae* LapX.** A. BLAST-based SSN (using the 10k blast option from ESI-EFT) at alignment score 130. The sixth largest cluster (cyan) is the cluster that contains LapX. B. Genome neighborhood diagram analysis. The labels indicate the Pfam name of the gene family located nearby. The larger the circle, the more genes in that category. None refers to genes that have not been assigned to any Pfam. Searching genomes of LapX-containing organisms reveals that a gene encoding an additional protease domain commonly resides nearby indicated by the relatively large size of the node. C. Related *Vibrio* species that show two different genomic architectures for proteases. Left: *Vibrio* species harboring two protease genes near *lapX*. Right: *Vibrio* species in which no obvious protease genes reside near *lapX*. Red denotes the S1 A peptidase domain (*lapX*), dark green is the M28 peptidase domain. Genes that reside near genes specifying proteases and that are oriented in the same direction are labeled with their bioinformatic annotations. The LapX gene is colored red in this diagram.


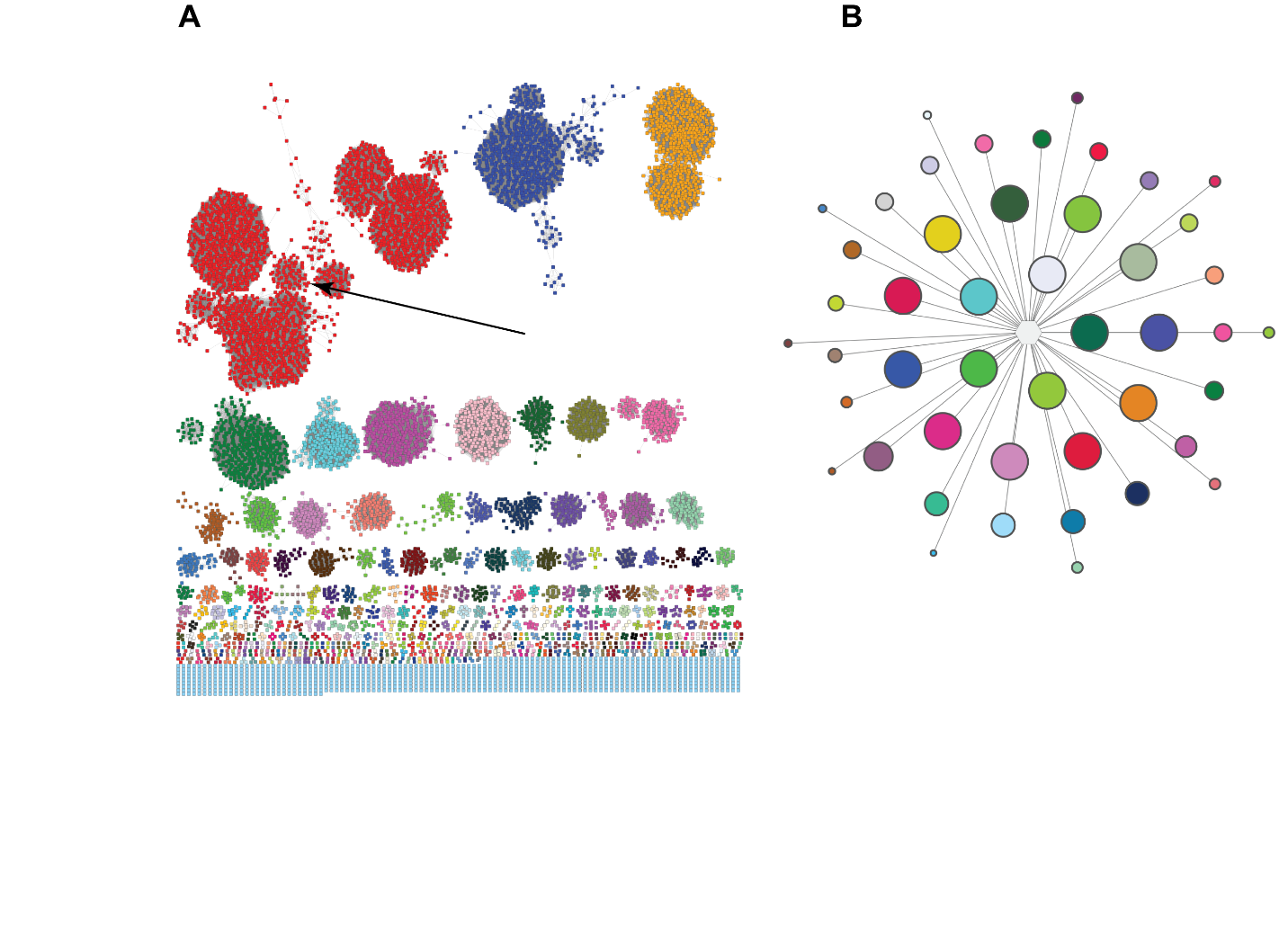


**Supplemental Figure 3. Sequence similarity network of proteases related to *V. cholerae* Lap.** A. BLAST-based SSN (using the 10k BLAST option from ESI-EFT at the alignment score 130). Lap is in the largest cluster (red). B. Genome neighborhood diagram analysis. A gene encoding the M28 protease is most commonly located near the *lap* gene. *lap* is also an M28 gene, but this analysis does not include *lap*. The circles and colors represent other M28 domain clusters from the network where every cluster that is present has two M28 genes residing in close proximity to one another in the genome; the larger the circle, the more M28 genes in that cluster.


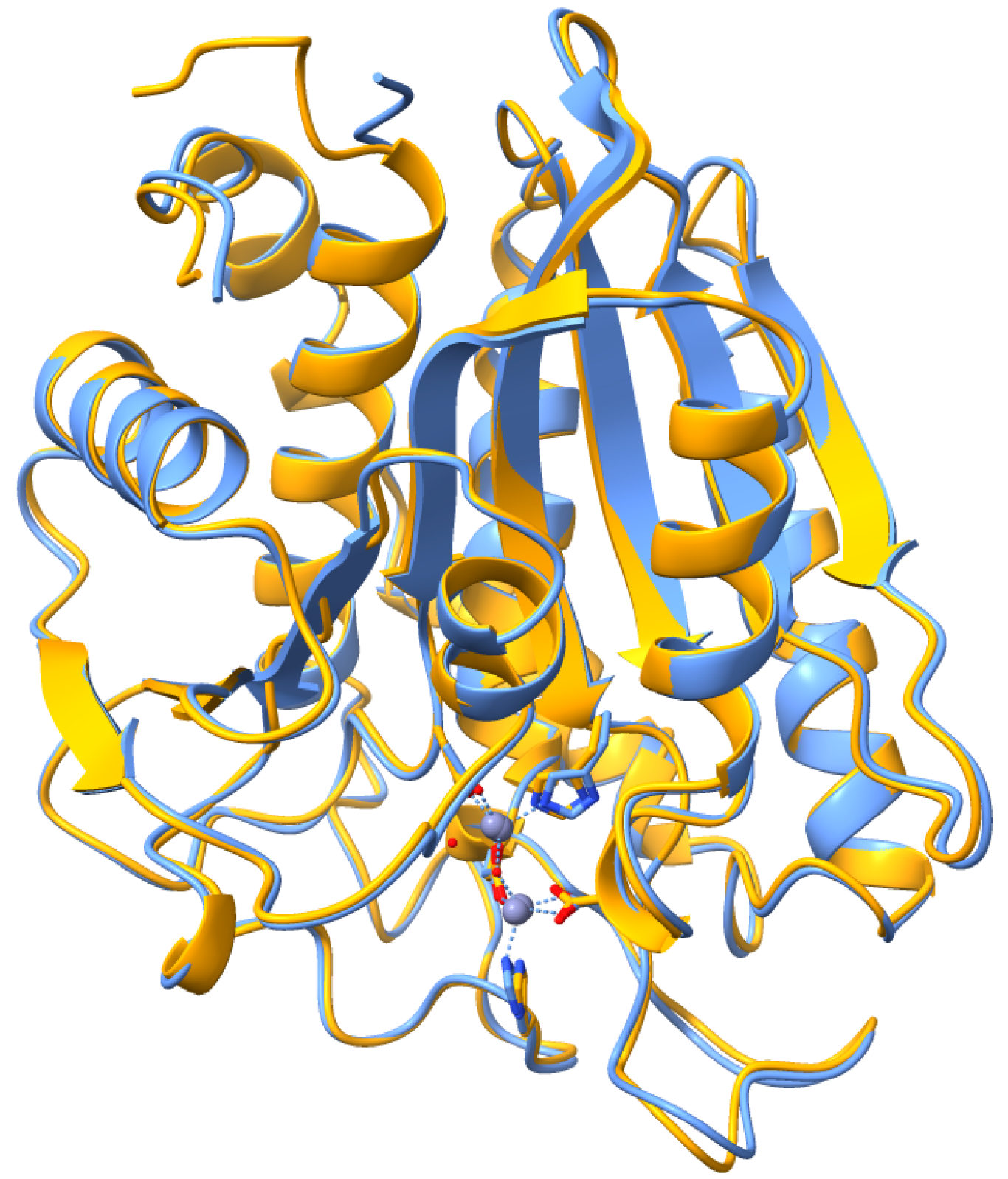


**Supplemental Figure 4. AlphaFold2 and AlphaFill models of *V. cholerae* Lap overlayed with *Vp*AP.** Overlay of the crystal structure of the protease *Vp*Lap (light-blue) (1AMP) with the *V. cholerae* Lap protease domain according to the AlphaFold2 and AlphaFill predictions (gold). Zinc atoms are depicted as purple spheres. The oxygen atoms (red) from water molecules located near the active site are shown.


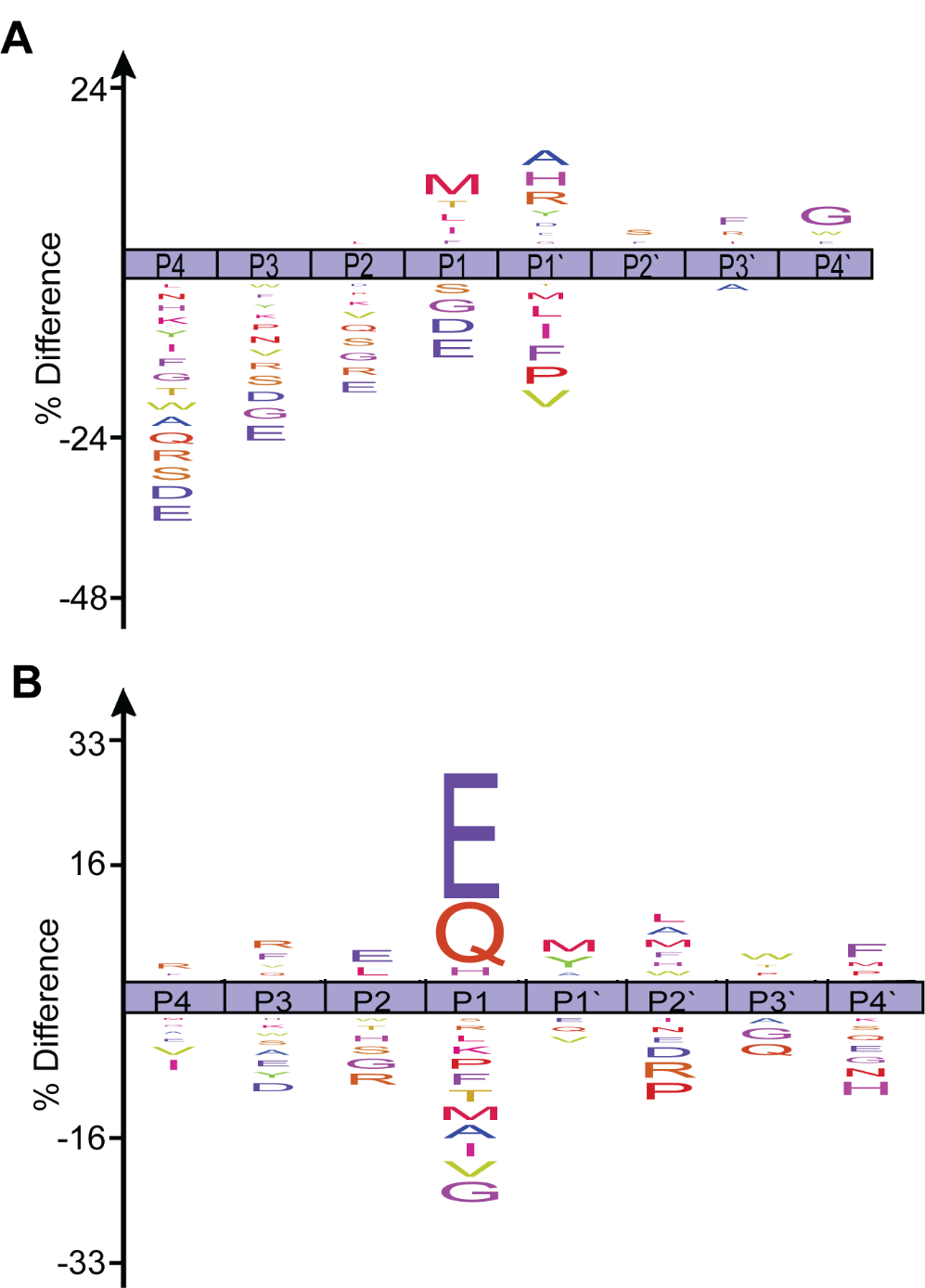


**Supplemental Figure 5. MSP-MS results for C-terminal truncated Lap and LapX.** A. ICE-LOGO of the MSP-MS results for C-terminal truncated Lap. Lap ΔPPC prefers Met, Thr, Ile, or Leu at the N-terminus. Lap ΔPPC prefers His, Arg, Ala, or Tyr at the P1` position. B. ICE-LOGO of the MSP-MS results for C-terminal truncated LapX. LapX ΔC is most active when Glu and Gln are at the P1 position. The percent difference refers to the relative abundance of this amino acid in its respective position when cleaved as compared to the entire library of peptides (1).


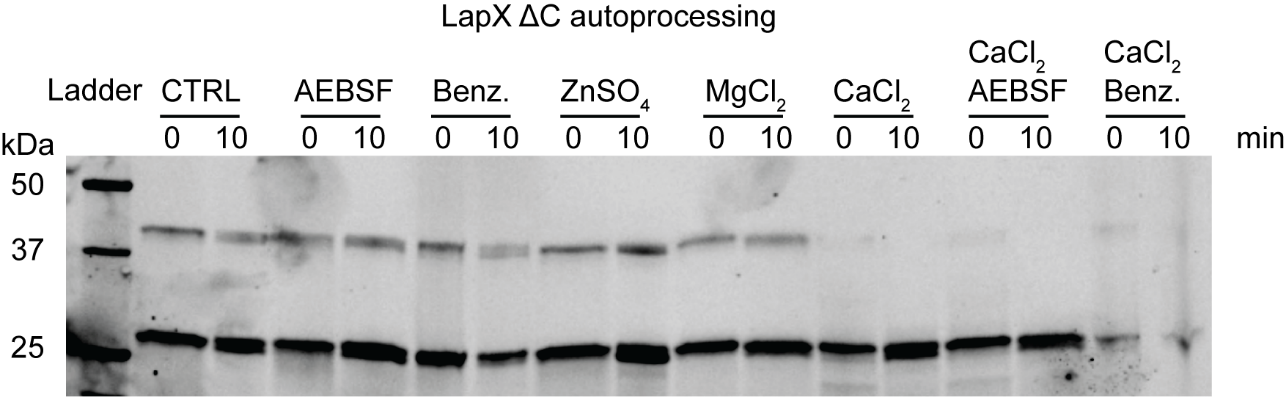


**Supplemental Figure 6. Effect of potential cofactors and inhibitors on LapX auto-processing.** Auto-processing by LapX ΔC was assessed by SDS-PAGE following the designated treatments. 10 µM of LapX ΔC in 50 mM TAPS pH 8.0 was treated with 1 mM AEBSF, 40 mM Benzamidine (denoted Benz.), 1 mM ZnSO_4_, 1 mM MgCl_2_, or 1 mM CaCl_2_ followed by incubation at RT for 10 min. The untreated control sample is labeled CTRL and was diluted to 10 µM in buffer and incubated for 10 min. For samples containing both CaCl_2_ and a protease inhibitor, LapX ΔC was pretreated with 1 mM AEBSF or 40 mM Benzamidine for 30 min at RT prior to addition of 1 mM CaCl_2_. Subsequently, samples were incubated at RT for 10 min. At times 0 or 10 min, as indicated, samples were treated with 5x SDS-loading dye to quench the reactions.


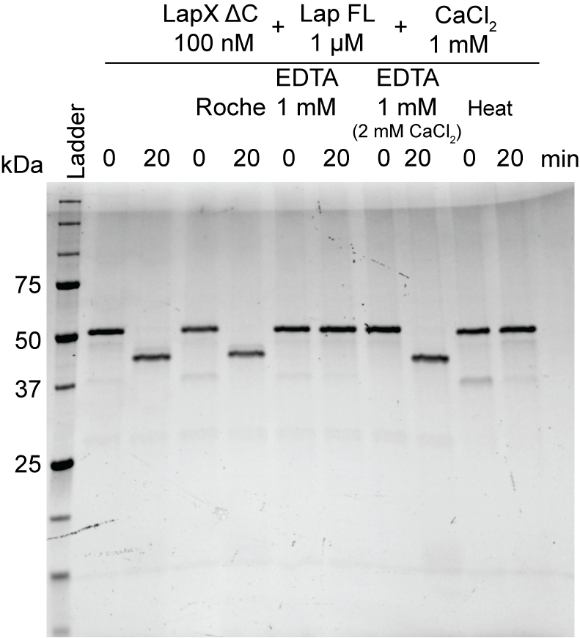


**Supplemental Figure 7. Inhibition of LapX processing of Lap.** Prior to the addition of 1 µM Lap FL, 100 nM LapX ΔC was pre-treated with 1 mM CaCl2 and an EDTA-free protease inhibitor cocktail (designated Roche) at the manufacturer’s suggested concentration for 30 min, 1 mM EDTA and 1 mM CaCl2 for 30 min, 1 mM EDTA and 2 mM CaCl2 for 30 min, or by boiling for 10 min with 1 mM CaCl2 (Heat). The untreated control sample is labeled CTRL. Lap is the upper band (apparent MW ~53 kDa) and is processed to the smaller band (apparent MW ~45 kDa). LapX ΔC processing of Lap FL (upper band MW ~53 kDa) is not affected by the Roche protease inhibitor cocktail but is inhibited by EDTA in the absence of excess calcium or by boiling.


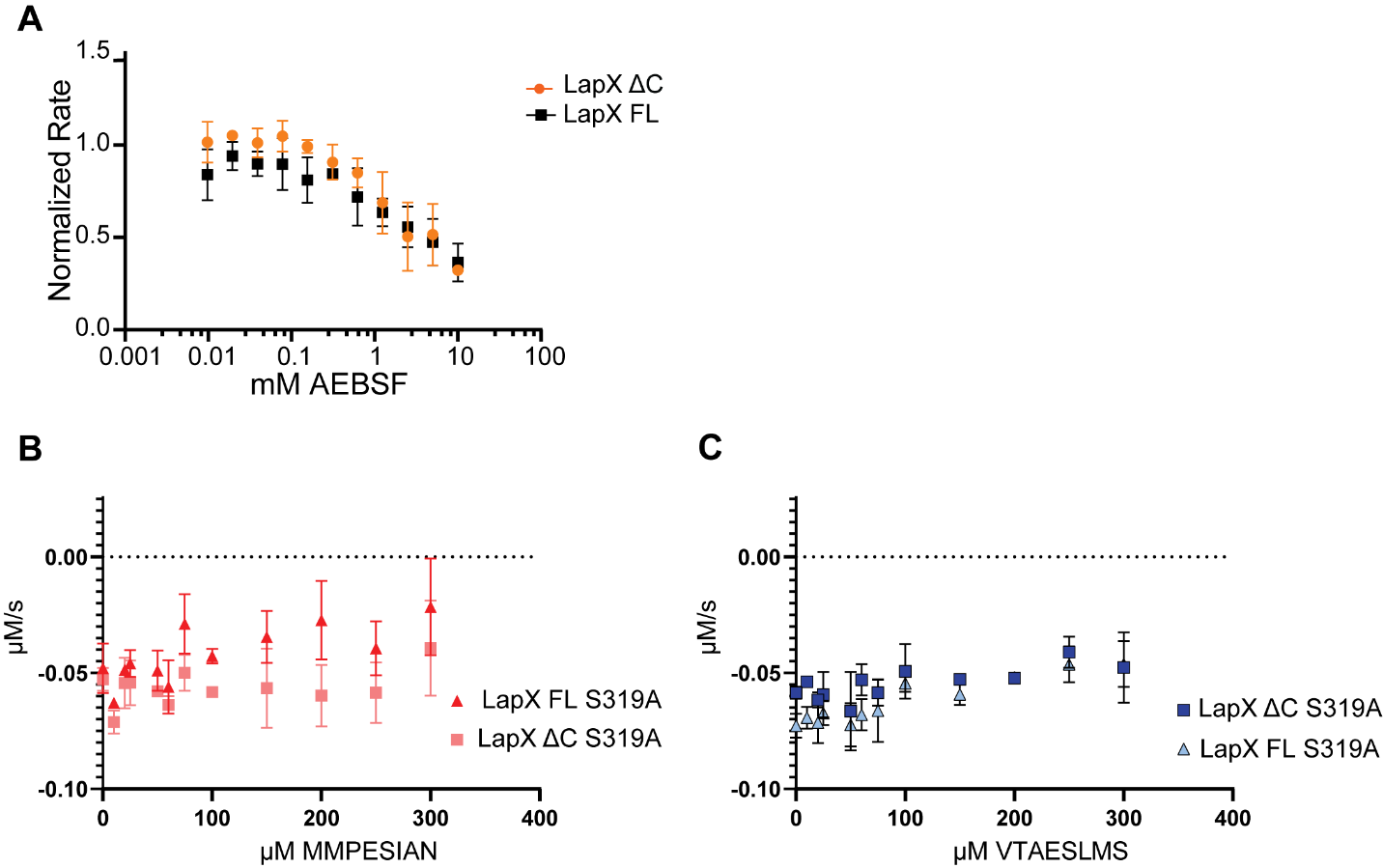


**Supplemental Figure 8. LapX enzyme kinetics with a physiologically based internally-quenched peptide substrate.** A. Inhibition curve of AEBSF on LapX. This curve shows AEBSF-mediated inhibition of LapX catalysis of the internally quenched FRET-based peptide substrate K(MCA)MMPESIAN K(DNP). Rates plotted were normalized to the rate without inhibitor present. B. Kinetics of LapX ΔC S319A and LapX FL S319A proteins. Shown are the activities of these proteins on the internally quenched FRET-based peptide substrate K(MCA)MMPESIAN K(DNP). Mutation of LapX serine residue 319 to alanine ablates LapX catalytic activity. C. Kinetics of LapX ΔC S319A and LapX FL S319A proteins. Shown are the activities of these proteins on the internally quenched FRET-based peptide substrate K(MCA) VTAESLMS K(DNP). Mutation of LapX serine residue 319 to alanine ablates LapX catalytic activity.


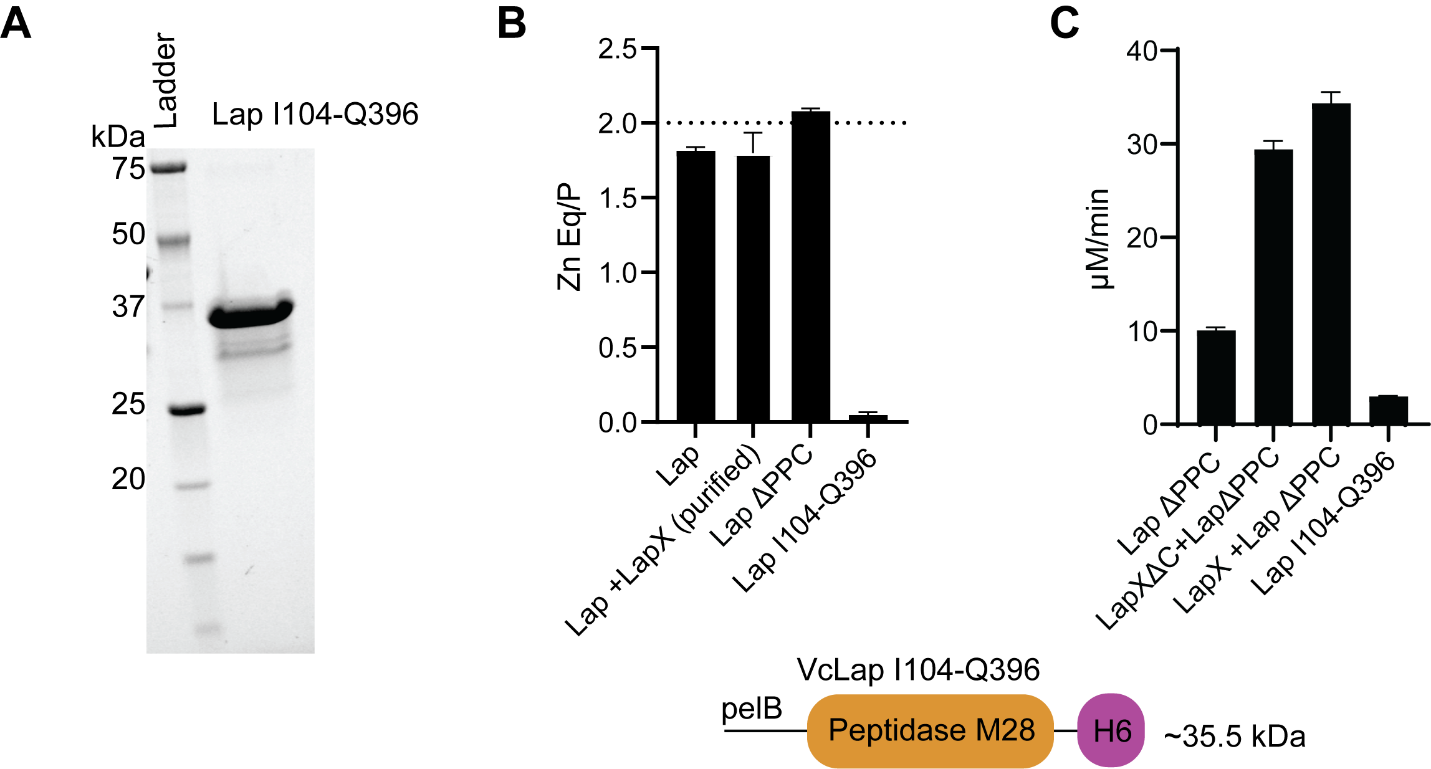


**Supplemental Figure 9. Purification of the Lap M28 domain (I104-Q396) and comparison of Zn content and activity to other Lap protein constructs**. A. Representative SDS-PAGE analysis of a sample of the Lap protein M28 domain (called Lap I104-Q396) following Zn^2+^-IMAC purification. B. Comparison of Zn^2+^ content of the designated Lap and LapX proteins. C. Activity on leucyl p-nitroanilide of Lap ΔPPC with or without co-incubation with LapX FL or LapX ΔC compared to the activity of the untreated Lap M28 domain (called Lap I104-Q396). The domain architecture of this construct is shown below panels B and C, and contains the pelB signal peptide, M28 domain, and poly-histidine tag (H6).


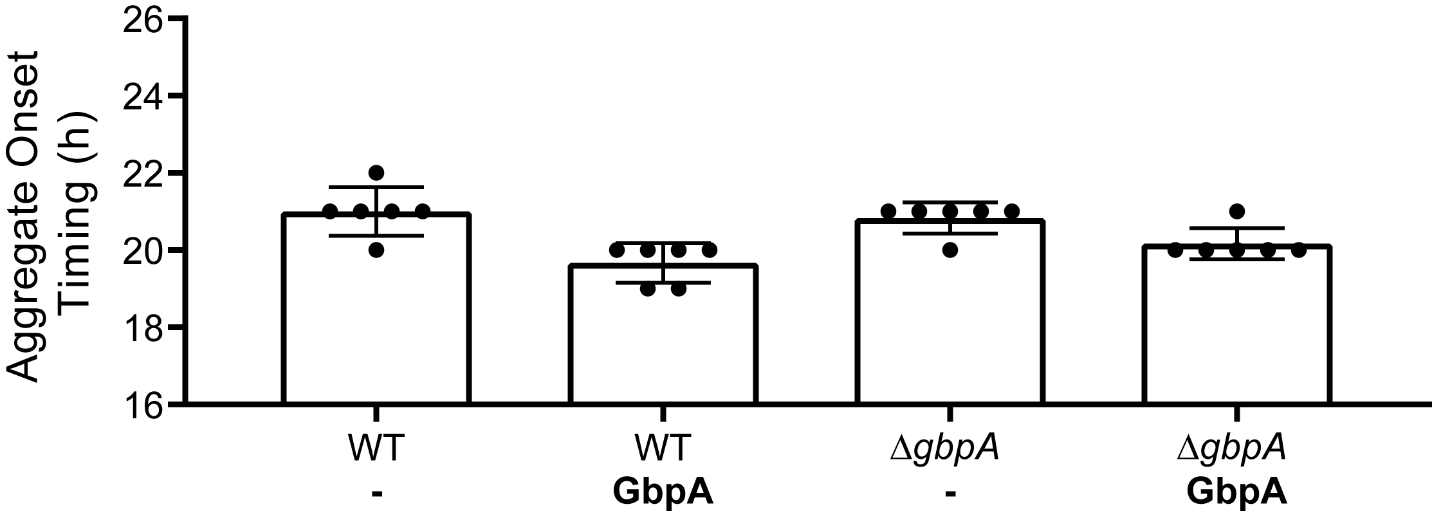


**Supplemental Figure 10. GbpA does not influence aggregation onset timing in *V. cholerae*.** Aggregation timing for the designated *V. cholerae* strains that did or did not have purified GbpA added.

**Supplemental Table 1.** Primers used in this study.

| **Primer name** | **5’ to 3’ primer sequence** |
| --- | --- |
| Lap FL | |
| *vca0813* F | ctgcccagccggcgatggccGAAGACAAAGTCTGGATCTCG |
| *vca0813* R | cagtggtggtggtggtggtgCTGAGTTGAGGCTTTTAACG |
| pET22B F | CGTTAAAAGCCTCAACTCAGcaccaccaccaccaccactgagatc |
| pET22B R | GAGATCCAGACTTTGTCTTCggccatcgccggctgggcag |
| Lap I104-Q396 | |
| Lap F | cccagccggcgatggccatgATCCCCGCACCTTCACAGCA |
| Lap R | tggctgccgcgcggcaccagTTGAGATGAGCCTGCATTGG |
| pET22B F | CCAATGCAGGCTCATCTCAActggtgccgcgcggcagcca |
| pET22B R | TGCTGTGAAGGTGCGGGGATcatggccatcgccggctggg |
| Lap ΔPPC | |
| Lap ΔPPC F | CACCACCACCACCACCAC |
| Lap ΔPPC R | GCGCTTCTGAGTGCCTCG |
| LapX FL from Genomic DNA | |
| *vca0812* F | cccagccggcgatggccCAGAGCACCGCTCCAACCATTG |
| *vca0812* R | gtggtggtggtggtgctcTTATTGTTTTTCAGTCGCAGTGAGTG |
| pET22B F | CAATGGTTGGAGCGGTGCTCTGggccatcgccggctggg |
| pET22B R | CACTCACTGCGACTGAAAAACAATAAgagcaccaccaccaccac |
| LapX ΔC | |
| LapX ΔC F | CACCACCACCACCACCAC |
| LapX ΔC R | TCTCACTTGAGGTGGCGTTTG |
| LapX FL S319A | |
| LapX S319A F | TGGCGGCTCTGCGGGCTCCCCCG |
| LapX S319A R | ATGGTGTCGCAGAAGTAGCCAGTGTCCGTGTGAGTG |

**Supplemental Table 2.** *V. cholerae* strains used in this work

| **Strain** | **Genotype** | **Reference** |
| --- | --- | --- |
| Vc0464 | Δ*vca0811* Δ*vpsL luxO* D61A *lacZ*::Ptac-*mKO* v*c1807*::KanR | Jemielita, et al., 2021 |
| Vc0465 | Δ*vca0812* Δ*vca0813* Δ*vpsL* *luxO* D61A *lacZ*::Ptac-*mKO* *vc1807*::KanR | Jemielita, et al., 2021 |
| Vc0466 | Δ*vca0812* Δ*vpsL* *luxO* D61A *lacZ*::Ptac-*mKO* *vc1807*::KanR | Jemielita, et al., 2021 |
| Vc0467 | Δ*vca0813* Δ*vpsL* *luxO* D61A *lacZ*::Ptac-*mKO* *vc1807*::KanR | Jemielita, et al., 2021 |
| Vc0572 | Δ*vpsL* *luxO* D61A *lacZ*::Ptac-*mKO* *vc1807*::KanR | Jemielita, et al., 2021 |

**References:**

1. O’Donoghue, A. J., Eroy-Reveles, A. A., Knudsen, G. M., Ingram, J., Zhou, M., Statnekov, J. B., Greninger, A. L., Hostetter, D. R., Qu, G., Maltby, D. A., Anderson, M. O., DeRisi, J. L., McKerrow, J. H., Burlingame, A. L., and Craik, C. S. (2012) Global Identification of Peptidase Specificity by Multiplex Substrate Profiling. *Nat Methods*. **9**, 1095–1100
